# A school-based intervention to improve mental health outcomes for children with cerebral visual impairment (CVI): feasibility cluster randomised trial

**DOI:** 10.1186/s40814-025-01603-x

**Published:** 2025-03-03

**Authors:** Cathy Williams, Anna Pease, Trudy Goodenough, Katie Breheny, Beverly Shirkey, Rose Watanabe, Parisa Sinai, Manmita Rai, Innes C. Cuthill, Mark Mumme, Andrew W. Boyd, Cassandra Wye, Chris Metcalfe, Daisy Gaunt, Kate Barnes, Siobhan Rattigan, Stephanie West, John Ferris, Jay Self

**Affiliations:** 1 Bristol Medical School, University of Bristol, Bristol, UK; 2 Department of Health Economics, University of Bristol, Bristol, UK; 3 Bristol Trials Centre, University of Bristol, Bristol, UK; 4 School of Biological Sciences, University of Bristol, Bristol, UK; 5 Population Health Sciences, University of Bristol, Bristol, UK; 6 UK Longitudinal Linkage Collaboration, University of Bristol, Bristol, UK; 7 authorsalouduk.co.uk, Bristol, UK; 8 Somerset NHS Foundation Trust, Taunton, UK; 9 University Hospital Southampton NHS Foundation Trust, Southampton, UK; 10 Cheltenham and Gloucester NHS Foundation Trust, Cheltenham, UK

**Keywords:** Cluster randomised trial, Feasibility trial, Cerebral visual impairment, Children, Special educational needs (SEN)

## Abstract

**Background:**

Cerebral visual impairment (CVI) refers to brain-related vision difficulties, which are often undiagnosed and may lead to poor mental health outcomes. We have developed an intervention to improve mental health outcomes for affected children, and it requires evaluation. The aim of this study was to assess the feasibility of methods proposed for a future definitive cluster randomised trial.

**Methods:**

This 18-month study took place in South West England, UK, between 2019 and 2021 including a 6-month pause due to the COVID pandemic. Participants were children aged 7–10 years in mainstream primary schools and their teachers and parents. We recruited head teachers on behalf of their school. The intervention was a resource pack for teachers explaining about CVI, providing universal and targeted strategies to help children with CVI and the offer of CVI assessments at the local eye clinic. The control schools continued with usual practice. Our objectives were to evaluate the feasibility of recruitment and data collection, attrition, acceptability of the study methods and implementation of the intervention. We conducted a process evaluation including interviews and questionnaires.

**Results:**

We sent invitation letters to 297 schools, received responses to 6% and recruited 40% of these (7 schools, 1015 children). Parents of 36/1015 (3.5%) children opted out. Baseline data were collected from teachers for 94% children, and 91% children completed self-report questionnaires; parent-report questionnaires were returned for 19% of children. During the exceptional circumstance of the COVID pandemic, two schools left the study, and many children were not attending school, meaning follow-up data were received from 32% of children, 16% of teachers and 14% of parents. Interview data indicated that the intervention was acceptable, and teachers would have preferred on-site eye tests to the offer of a clinic appointment and a clear timetable for study events. Teachers in intervention schools reported expected changes in the children’s and their own behaviour. There was some contamination between study arms.

**Conclusions:**

A full-scale trial would be feasible, enhanced by insights from this feasibility trial, in non-pandemic times. Sharing these data with teachers, education policymakers and parents is planned to refine the design.

**Trial registration:**

ISRCTN13762177.

**Supplementary Information:**

The online version contains supplementary material available at 10.1186/s40814-025-01603-x.

## Key messages regarding feasibility

- What uncertainties existed regarding the feasibility?
  As no previous school-based cluster randomised trials have involved an intervention relating to children’s visual impairments, we wanted to know whether a trial combining school-based and eye clinic-based elements would be feasible and acceptable. We wanted to know whether we could collect cost data from parents and schools related to special educational needs and disability (SEND). We also wanted to know whether we could link the study children’s data to administrative data from the Department for Education (DfE) that could provide additional outcomes. Finally, we wanted to know whether the new intervention was acceptable and how it was implemented.
- What are the key feasibility findings?
  Few (6%) responses to the unsolicited invitation letters were received, but enrolment after telephone calls was 40%. Usable baseline data were obtained for 94% children (from teachers) and 91% children as self-report, using the proposed outcome measures; however, the completed questionnaires from individual schools varied between 79 and 100%. Fewer data were received from parents, relating to 19% of children at baseline. Offering a voucher increased the number of follow-up responses from parents in two schools. The COVID pandemic necessitated changing the offer of a clinic referral to the offer of a telephone call and led to reduced follow-up data as most children were not attending school and two schools left the study. Four of seven schools returned questionnaires giving costs related to SEND at baseline and three at follow-up. The intervention was acceptable — however, teachers expressed a preference for on-site CVI assessments rather than referrals to a clinic. Teachers reported expected changes in their own and the children’s behaviour. A high proportion (97.5%) of the study children were linked to their DfE data.
- What are the implications of the feasibility findings for the design of the main study?
  A different approach to publicising the study may be better for recruitment; data returns from each school (cluster) should be monitored, parent questionnaires should be shorter, vouchers were offered as a thank-you and schools need a clear timeline of events. The intervention should include on-site vision assessments rather than referral to a clinic. Study team members should take classroom photographs and assist schools with data collection if wanted. Linkage of study children to DfE data is feasible and could provide costs incurred by schools supporting children with SEND.

## Background

Cerebral visual impairment (CVI) is an umbrella term referring to a group of visual difficulties or impairments due to abnormalities in the central parts of the visual system, such as an inability to see a target amidst clutter or to recognise people and objects especially from unusual angles, problems with route finding and difficulties making accurate visually guided movements [1, 2]. In children, these can occur after damage to the central visual pathways in perinatal or childhood illness [3, 4] or in the context of genetic [5–7] or behavioural neurodevelopmental conditions [8].

There is not yet agreement on the exact thresholds or criteria that should be used to diagnose CVI [2], and many vision tests are used [9, 10]. However, there is broad agreement that children at risk of CVI should be assessed with age-appropriate tests to identify CVI-related vision problems, and if these are present, for them to be supported by a range of professionals [11, 12].

In a recent survey in mainstream primary schools in England, our group reported that CVI-related vision problems were more prevalent than has been appreciated, affecting 3.4% (95% *CI*: 2.5 to 4%) of all participating children and 41.7% (95% *CI* 33.5 to 50.2%) of children tested who were having additional help due to recognised special educational needs (SEN) [13]. Another study reported a quarter of children being educated in a special school (a school specifically for children with SEN) and had vision processing problems [14]. Although visual acuity screening in reception class (aged 4–5 years) is recommended in the UK [15], many children with CVI-related vision problems have good visual acuity (85% in our recent study) [16] and are therefore not identified by acuity-based vision screening. The impact of CVI-related vision problems varies between children: some need extensive support, whilst others need little help and/or find their own strategies to cope [17]. Families of some children with CVI reported in interviews that their children had developed frustration and anxiety before their CVI was identified, leading to frequent “meltdowns” and low self-esteem, and that simple strategies at school and at home could help the children achieve their tasks better [17].

We developed an intervention that aims primarily to improve mental health outcomes for children with CVI-related vision problems in mainstream primary schools, whether diagnosed or not, and this requires evaluation. Following the MRC guidance [18], we have carried out a feasibility cluster randomised trial (cRCT), using schools as the clusters and including a process evaluation (PE). We also explored the feasibility of collecting data to support a future health economic evaluation. The methods have been published in full elsewhere [19, 20]. Here, we report the results, following the CONSORT extension for feasibility and pilot studies [21] and the recommendations of a recent review on reporting school-based feasibility studies for cluster randomised trials [22].

### Aims

Our aims were to investigate the feasibility of a future, definitive cluster randomised trial with an embedded health economic evaluation, evaluating a new intervention designed to improve mental health outcomes in primary school children with CVI-related vision problems. To achieve this, we conducted an external pilot study with random allocation of schools, following the proposed protocol for the full trial.

Our main objectives were to evaluate the feasibility of each method we proposed for the definitive future trial, to review data that could inform the selection of the primary outcome in the definitive trial and to investigate the implementation and acceptability of the intervention. Additional objectives were to evaluate two adaptations of existing methods for potential use in the future definitive trial. These were (i) to explore whether teachers’ and parents’ responses were similar, when asked to complete a simple CVI screening tool (the Five Questions, 5Qs) [23], and (ii) using a software programme to analyse classroom photographs and thereby derive a quantitative estimate of visual clutter in the children’s environment, for use as a measure of intervention implementation [24, 25].

## Methods

### Trial design

This was a feasibility cluster randomised trial. We randomised at school level, and we used a 1:1 allocation ratio. The outcome measures assessed several indicators of a child’s wellbeing and/or mental health: child self-reported health-related quality of life (HRQoL) using the PEDSqL [26], teacher-reported child behaviour (Strengths and Difficulties Questionnaire) [27]and cognitive abilities [28] and parent-reported HRQoL and family functioning (Family Impact Module) [29]. SEN-related cost data were collected from schools by a questionnaire to the key contacts: from parents using a bespoke questionnaire on service use and time spent supporting their child’s health or education, and we planned to collect resource use data from the eye clinic in interviews and surveys to staff. Costs were estimated using NHS reference costs and PSSRU (Personal Social Services Research Unit) Costs for Health and Social Care. CHU9D utilities were estimated by mapping from the PedsQL Core Scales using the algorithm reported by Lambe et al. [30] The definitive trial is planned to include linkage to pupil’s routine attainment and attendance records from the Department for Education (DfE), so an application was made to link the data from this feasibility trial to variables in pupil-level and school-level datasets in the National Pupil Database (NPD). A process evaluation (PE) collected information on the feasibility trial processes and on the intervention using interviews (teachers and parents), questionnaires about knowledge of CVI [31], self-efficacy [32] and document review of the school websites. The feasibility trial began in September 2019 and was due to finish in July 2020. We aimed to use the learning from the PE, as well as the quantitative data, to decide whether to proceed with a future trial using these data and advice of the Trial Steering Committee (TSC), rather than set progression criteria.

### Eligibility criteria and setting

Children in mainstream primary schools years 3–5 (ages 7–10 years) were eligible for inclusion if they were in one of the three study areas (Southampton, Gloucester and Somerset) where the participating paediatric ophthalmologists worked. Schools were excluded if they shared a Special Education Needs Co-ordinator (SENCo) with another participating school, to avoid potential contamination between schools in different arms of the study.

### Recruitment and consent

We sent out postal and email invitation letters to all eligible schools in the summer of 2019, asking interested schools to contact us. We spoke to schools that got in touch and sent study documents if requested. If the head teacher signed a memorandum of understanding (MOU), the school was recruited. Participant information sheets (PIS) were sent to all parents and teachers including an “opt-out” slip for parents which if returned to us meant we deleted the child’s study ID and did not include their data. Parents and teachers who were interviewed gave written informed consent.

### Intervention

The intervention used the framework of “proportionate universalism” [33] (a framework proposed as an approach to reducing childhood health inequalities) and aimed to increase teachers’ awareness of CVI and give them strategies they could use to help, thereby improving affected children’s school experience. It comprised a PowerPoint presentation about CVI sent to the school key contact by email: a plastic box for each class containing (a) a written transcript of the PowerPoint; (b) two laminated advice sheets, one with “universal” interventions to reduce visual clutter in the school environment and one with “targeted” interventions for specific children who were struggling with their learning; (c) letters for a parent to give to a child’s GP, requesting referral to the local paediatric ophthalmology clinic to be assessed for CVI, for up to 5% of children (a limit set arbitrarily to avoid over-burdening GPs and clinics); (d) a sheet with guidance on which children are at higher risk for CVI and might benefit from a referral; and (e) sheets with vision-related stories and activities for the teachers to use with children, linked to the school curricula. The control intervention was “care as usual”, and no extra materials were provided.

### Changes in the design

The feasibility trial started in September 2019 and was paused in March 2020 when all UK schools were closed because of the COVID pandemic [34]. Over the next year until March 2021, school life was extremely disrupted. There were prolonged periods when only children who were vulnerable (known to the social care services and/or had an Educational Health and Care Plan) or were the children of key workers (such as health or food distribution workers) were able to attend school, and the majority of children were educated at home. There were periods of local variations in restrictions, according to prevailing infection rates [34].

We adapted the design of the feasibility trial and restarted in September 2020. The changes were as follows: the study was extended until March 2021, the offer of a visit to the eye clinic was changed to the offer of a CVI telephone assessment, and teachers could opt-out of the follow-up questionnaires, and the parent and child follow-up questionnaires could be completed online unless the child was attending school. Vouchers were offered as incentives for parents to complete their follow-up questionnaires.

### Specific outcomes for the feasibility trial

Our outcomes were to answer the research questions presented in Table 1, together with the methods we used to address each question.

**Table 1:** Box showing feasibility study outcomes and how each was assessed

Number	Outcome domain	How assessed
1	Recruitment response	% schools responding
2	Parents asking for opt-out	% parents returning opt-out slip
3	Attrition of schools (clusters) and children	% schools (clusters) and/or children leaving the study
4	Yield from outcome measures	% questionnaires with usable data
5	Data to inform sample size calculation for future trial	Mean (standard deviation, SD) or median (interquartile range, IQR), range, intraclass correlation coefficient (ICC)
6	Feasibility of linkage to DfE data	% children and schools linked to DfE data and with usable data
7	Feasibility of collecting SEN-related costs from school, parents and eye clinic	% returned questionnaires with usable data
8	Acceptability of study methods to parents and school staff	Interviews with teachers and parents
9	Implementation measures including fidelity, dose, adaptation, reach, sustainability	Questionnaires to school contacts, interviews with teachers, number children referred to eye clinic, document review of school websites
10	Mechanisms of impact	Interviews with school contacts, questionnaires for teachers on (a) CVI knowledge and (b) self-efficacy
11	Acceptability of intervention	Interviews with teachers and parents
12	Comparison between teacher and parent responses to the Five Qs screening questions	% of children for whom parents and teachers’ responses indicate higher risk for CVI
13	Feasibility of using classroom photographs to provide objective measure of visual clutter	% of schools returning photographs and % with usable data

### Harms

These are any unexpected or adverse effects noted in the data from the interviews or recorded in reports to the sponsor.

### Feasibility study sample size

The primary aims of this study were to gain experience in, refine and consider the feasibility of the recruitment, intervention delivery and outcome assessment procedures for a future definitive cluster randomised study in a resource-efficient study, and we judged that this would be achieved with eight schools, with at least four schools receiving the intervention. It has been argued that feasibility studies will be too small to estimate quantitative parameters of interest for the design of a cluster randomised trial such as the ICC [35].

### Randomisation

An independent statistician randomised the schools using a 1:1 ratio, after baseline data were collected, with stratification by school size (1–2 classes per year vs 3 +) and proportion of recruited children with special education needs and disabilities (SEND, less than 15% vs 15% +).

### Masking

With this universal intervention, it was not possible to mask teachers or parents to which arm of the study they were in. The study team were also not masked to the group allocations, although the study statistician was not aware of which arm received the intervention whilst conducting the analysis.

### Analysis

We used counts and percentages with 95% confidence intervals (CI) for key estimates, to summarise recruitment, retention, data completion and linkage to DfE administrative data. Characteristics of outcome data were described with means (standard deviations, SD), medians (interquartile ranges, IQR), ranges, intraclass correlation coefficients (ICC) and mean estimates of change with 95% confidence intervals. The ICCs were calculated using the “estat icc” command after mixed-effects regressions of the outcome scores including class and schools as random effects in *Stata* 18™. Interviews were recorded with permission, and transcribed and thematic analysis was used to summarise and interpret the data. The responses from key contacts to a short questionnaire about how they used the intervention are presented descriptively. The photographs were analysed using an image processing algorithm to derive summary measures of visual clutter. We used a measure of visual clutter known as “feature congestion” [24, 25, 36], which has been shown to predict the difficulty of visual search for targets in complex scenes [24, 25, 36]. The feature congestion metric was calculated using the publicly available MATLAB [37] functions of Rosenholtz et al. [24, 38]. Briefly, feature congestion measures the amount of variation in a scene using three components of early visual processing: luminance contrast, chromatic contrast and edge orientation. It does so at multiple spatial scales and then combines them, using empirically derived weights, in a single measure. A scene with lots of variation in brightness (dark and light objects), hue (different coloured objects) and the orientation of lines (e.g. the boundaries of objects such as sheets of paper pinned to a board) will have a high measure of feature congestion. This metric was then compared between classroom photographs at baseline and follow-up, in boxplots.

The teachers’ responses to questionnaires about CVI and self-efficacy were collected at baseline and follow-up, and the mean (SD) change in scores is presented for each study arm. The school documents at the start and end of the study were compared in a narrative synthesis.

## Results

### Participant flow

**Fig. 1:**
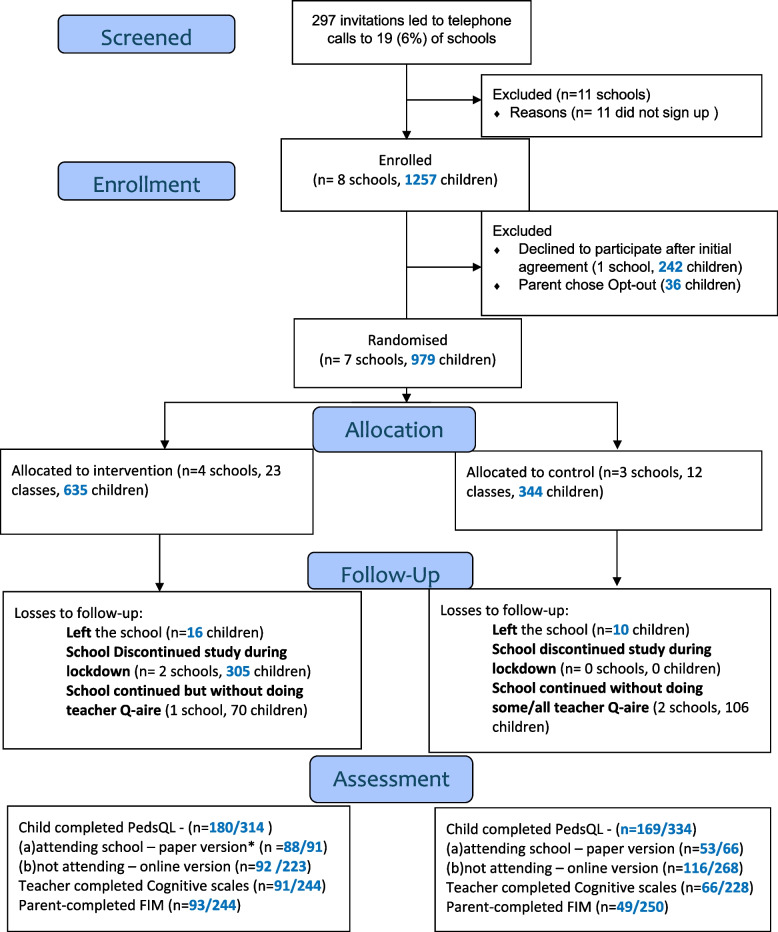
Flow chart showing numbers of participants at different stages of the trial

The flowchart in Fig. 1 summarises the participant numbers at each stage of the trial.

### Characteristics of participants

Table 2 summarises the characteristics of included children. They were evenly distributed between years 3, 4 and 5 with similar numbers of boys and girls. The majority (90%) were White; 15% had free school meals (an indicator of low household income), and 15% were having SEN support of some level. The arms were similar for the stratification variables but differed as regards to children having free school meals: 8.4% in control arm and 19.4% in intervention arm.

**Table 2:** Characteristics of 979 children in the 7 participating schools at baseline

	**School**							**Control** ***N*** ** (%)**	**Intervention** ***N*** ** (%)**	**Total** ***N*** ** (%)**
	**1**	**2**	**3**	**4**	**5**	**6**	**7**			
**Sex**										
**Boys**	47	95	33	44	35	137	101	177 (51.5%)	315 (49.6%)	492 (50.5%)
**Girls**	35	83	37	42	49	123	118	167 (48.5%)	320 (50.4%)	487 (49.7%)
**School year**										
**3**	28	58	29	29	30	87	58	116 (33.7%)	203 (32.0%)	319 (32.6%)
**4**	22	59	24	27	24	88	82	105 (30.5%)	221 (34.8%)	326 (33.3%)
**5**	32	61	17	30	30	85	79	123 (35.8%)	211 (33.2%)	334 (34.1%)
Ethnicity*										
**White**	78	164	63	58	77	256	189	319 (92.7%)	566 (89.3%)	885 (90.5%)
**Other**	4	14	7	28	7	4	29	25 (7.3%)	68 (10.7%)	93 (9.5%)
Free school meals*										
**No**	72	166	53	63	77	215	180	315 (91.6%)	511 (80.6%)	826 (84.5%)
**Yes**	10	12	17	23	7	44	39	29 (8.4%)	123 (19.4%)	152 (15.5%)
**Any SEN support**										
**No**	70	157	51	62	66	232	189	293 (85.2%)	534 (84.9%)	827 (84.5%)
**Yes**	12	21	19	24	18	28	30	51 (14.8%)	101 (14.9%)	152 (15.5%)
**Mean (range) classes per year**								1.3 (1–2)	1.9 (1–3)	

^*^One child with no data in each row

### Results for the feasibility outcomes

A full report of the process evaluation findings is included as Supplementary Material File 1, detailed descriptions of the data collected in the questionnaires are presented in Supplementary Material File 2 and parent responses in the heath and care resource questionnaires are given in Supplementary File 3. Below we summarise the main results to our research questions.

1. Recruitment
  We sent invitation letters and emails to 297 schools. We were approached by 19 (6.4%) of these schools, and after telephone calls with each, we enrolled 8 schools (1257 children, 42% of schools). One school dropped out after less than a week and before randomisation, so we finished recruitment with 1015 children in 7 schools.
2. Parents opting-out their children
  Thirty-six children (36/1015, 3.5%) were withdrawn from the study after their parents returned signed opt-out slips.
3. Retention
  Twenty-six children (16 in intervention schools and 10 in control schools) left their schools (26/979, 2.7%) during the study. Two schools (both in the intervention arm) comprising 305 (305/979, 31.2%) enrolled children and withdrew from the study during the COVID school closures. Of those remaining in the study, three schools (one in intervention and two in control arm) agreed to continue if their teachers did not have to complete follow-up questionnaires (relating to 176 children, 176/674, 26.1%).
4. Yield of data from outcome measures proposed for the future trial
  Table 3 summarises the proportion of completed questionnaires returned for each participant group’s outcome measures.

**Table 3:** Percentage data completion at baseline and follow-up for teacher, child and parent outcome measures proposed for use in future definitive trial, overall and by school (cluster)

School (cluster)	*N*	% questionnaires retuned with usable data					
		**Baseline: pre-pandemic**			**Follow-up during pandemic —-school closures**		
	**No. of recruited children**	**Teacher-report SDQ, cognitive scales**	**Child-report HRQoL** **Overall score**	**% parent-report Family Impact Module**	**Teacher-report SDQ, cognitive scales**	**Child-report HRQoL (paper + online)**	**Parent-report Family Impact Module**
**1**	82	79.3	92.7	17.1	20.7	51.2	24.4
**2**	178	100	96.1	21.3	27.5	22.5	11.8
**3**	70	85.7	93.0	10.0	0	91.4	0
**4**	86	77.9	88.6	0	n/a	n/a	n/a
**5**	84	92.9	94.1	27.4	0	94.1	0
**6**	260	100	96.5	26.9	35.0	36.9	36.9
**7**	219	96.8	79.5	16.0	n/a	n/a	n/a
**Cluster mean**	139.9	90.4	91.5	17.0	16.6	59.1	14.6
**Cluster SD**	77.9	9.4	5.9	9.7	16.0	32.3	16.0
**% (95% CI) of children recruited (979)**		94.0 (92.3 to 95.5)	91.2 (89.3 to 92.9)	19.1 16.7 to 21.7)	16.0 (13.8 to 18.5)	32.8 (29.9 to 35.8)	14.0 (11.9 to 16.6)

*n/a* schools that withdrew during study pause

Counting the children individually, 94% of teacher questionnaires and 91% of child questionnaires were returned; however, the proportions returned from each school (cluster) varied, and two schools returned less than 80% of their teacher questionnaires and one less than 80% of their child questionnaires. Returns from parents in every school were low at baseline, with individual schools returning between nil and 27.4% of their parent questionnaires.
  Follow-up questionnaire completion was reduced by many children not being in school so the teachers could not complete questionnaires about them. Children attending school completed paper questionnaires, and for children not at school, an online version of their questionnaire was attached to their parents’ online questionnaire. The proportion of completed child-report questionnaires (paper and online versions combined) received at follow-up from each school varied between 26.4 and 91.7%. The mean proportion of parent-completed questionnaires returned increased slightly at follow-up, possibly associated with the vouchers we introduced as an incentive, but the highest response proportion from a school was only 36.9%.
5. Characteristics of potential primary outcomes in the future trial, to inform sample size calculation
  Descriptions of baseline data and changes over the study are given in Table 4.

**Table 4:** Descriptives of data collected with outcome measures proposed for use in future definitive trial

Outcome measure (N)	Mean (SD) at baseline	Median (IQR) at baseline	Mean (SD) at FU	Median (IQR) at FU	Mean change (SD) FU-BL	BL ICC (95% CI) school	BL ICC (95% CI) class
Child-report HRQoL (PEDSQL)	72.6 (16.8)	75.0 (62.4 to 85.4)	70.4 (16.2)	71.7 (60.3 to 82.3)	− 0.8 (15.6)	0.04 (0.00 to 0.24)	0.16 (0.09 to 0.26)
Teacher-report total difficulties (SDQ)	6.7 (6.7)	5.0 (1.0 to 10.0)	6.7 (7.0)	4 (1 to 10)	− 0.4 (4.7)	0.03 (0.00 to 0.29)	0.12 (0.07 to 0.22)
Teacher-report impact score (SDQ)	0.6 (1.3)	0.0 (0.0 to 0.0)	0.6 (1.3)	0 (0 to 0)	0.0 (1.1)	0.01 (0.00 to 0.90)	0.05 (0.02 to 0.12)
Teacher-report cognitive scales (PEDSQL)	69.9 (27.4)	75.0 (50.0 to 100.0)	66.7 (28.9)	66.7 (50 to 95.8)	0.8 (24.1)	0.02 (0.00 to 0.32)	0.11 (0.06 to 0.20)
Parent-report impact score (SDQ)	0.9 (1.9)	0.0 (0.0 to 0.0)	0.9 (1.8)	0.0 (0 to 1)	0.0 (1.6)	N/a	N/a
Mean health and social care costs per family in last year (£)	489.2 (988.3)	170.1 (98.5 to 420.0)	308.6 (727.0)	93.4 (49.3 to 280.8)	2.7 (1144.8)	N/a	N/a
Mean hours in last month supporting child’s education or health	3.6 (16.5)	0.0 (0.0 to 1.0)	38.7 (128.7)	0.4 (0 to 8)	36.0 (114.1)	N/a	N/a
Child utility scores mapped from PedsQL	0.91 (0.06)	0.99 (0.88 to 0.96)	0.91 (0.05)	0.92 (0.87 to 0.95)	0.0 (0.06)	N/a	N/a

Footnote: For child-report PedsQL and teacher-report cognitive scales, higher score is better QoL or cognitive ability, and range for each is 0–100. For the SDQ, total difficulties (range 0–40) and impact scores (range 0–9 for teacher report, 0–15 for parent report), a higher score denotes more difficulties or examples of problematic behaviour

The mean and/or median values are in keeping with reports from similar samples of children [39–41]. There were no ceiling or floor effects, and individual children changed in either direction during the trial. The ICCs are larger when the cluster was the child’s class, for both teacher and child reports, than when the cluster was the school.
6. Feasibility of linking the participants’ study data to administrative data held by DfE
  We applied to link the study to selected DfE data once ethical committee permission was obtained, in summer of 2021. The older children had left their primary schools by then, so we gave parents the opportunity to opt their child out of this linkage to DfE data by sending emails with a parent information sheet (PIS) to the participating schools and to local secondary schools asking them to send the PIS to the parents of children who had joined in Y7. We also put notices with the PIS into a free magazine distributed to all primary school children, in each of the study areas and on our study website. No responses or queries were received relating to the linkage of the study to the DfE administrative data.
  The proportion of participating children linked to the DfE data was 955/979 (97.5%): 620/635 (97.6%) of the intervention group and 335/344 (97.4%) of the control group. Attendance, attainment and demographic data were available for nearly all the children. For example, attendance data in the school years 2018–2019 and 2020–2021 were available for 947/955 (99.2%) children: level of development in the early years foundation stage for 940/955 (98.4%) and key stage 1 results at age 6 years for 955/955 (100%). Financial data including yearly spend on SEN provision and on extra staff brought in to support pupils were available for all but one school, which did not feature in the financial tables. Further enquiry has established this was an academy school, and these do not submit financial data to DfE. Full results from analysing these data will be presented separately.
7. Feasibility of the SEN costs data collection methods
  The Health and Social Care (HSC) costs incurred by each family in the last 12 months (estimated using standard HSC item costs mapped to the parent/carer’s reports of service use) showed large variations between families, as shown in Table 4. On average, families reported more time in the last month spent supporting their children’s health or education at the end of the study, with similarly large variations. Child-based health utility estimates, mapped to the CHU9D, were similar at baseline to those reported in other studies using the same approach [42] but higher than directly elicited CHU9D values obtained in other trials with similar-aged children [43].
  Just over half (4 of 7, 57.1%) of schools returned their baseline questionnaires on the SEN-related costs they had incurred in the 2018–2019 school year, and three of these also returned one at follow-up. Summaries of the baseline responses are shown in Table 5.

**Table 5:** Data relating to costs incurred by schools related to special educational needs (SEN) support for students in years 3–5 in 2018–2019 school year, from the four schools who returned the SEND costs baseline questionnaire

School code	No. of classes in years 3–5 combined	Number of children (total contacts for all children) receiving support								LSAs
		**EP**	**Physio**	**OT**	**SaLT**	**CAMHS**	**Behav**	**Art**	**Play**	
1	3	2 (3)	0	0	2 (15)	0	1 (12)	1 (6)	2 (5)	4.0
2	6	1 (1)	2 (2)	2 (2)	1 (4)	0	1 (3)	1	0 (30)	19.6
3	3	1 (1)	0	1 (1)	2 (4)	0	0	0	0	0.0
5	3	4	0	1	2	2	1	0	0	8.0

Abbreviations: *EP* educational psychologist, *Physio* physiotherapist, *OT* occupational therapist, *SaLT* speech and language therapist, *CAMHS* Child and Adolescent Mental Health Services, *Behav* behavioural support, *Art* art therapist, *Play* play therapist, *LSA* learning support assistants

**Fig. 2:**
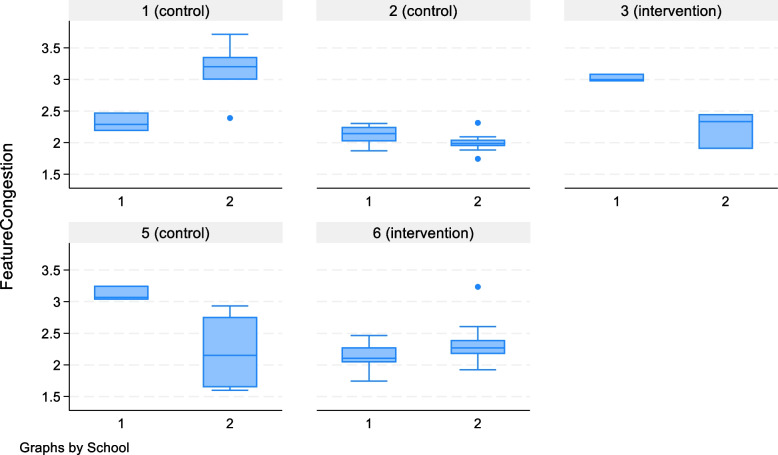
Boxplots of feature congestion scores in classroom photographs (>1MB) showing the front of the classroom, at the baseline and a follow up, by school

There was variation in the number of learning support assistants (per class and per school): in the numbers of contacts pupils had with external specialists and in the sources of funding used to pay for these. For example, three schools paid for educational psychology (EP) from their own budgets and one used “top-up” funding. Visits from occupational health (OT), physiotherapists (PT), speech and language therapists (SALT) and child mental health services (CAMHS) practitioners were funded by the local health service in two schools, top-up funding from the local authority in one school and the school SEN or pupil premium budgets in another school. No costs were collected from eye clinics.
8. Acceptability of the study methods to teachers and parents
  Seven teachers and 16 parents were interviewed. In the two schools that withdrew during the COVID closures, one contact commented that “there was a lot to do” for this trial, and that was a factor in their decision, whilst the other contact gave no reason for their withdrawal. All contacts interviewed reported that completing the questionnaires was not problematic either for the teachers or the children, and that the money we reimbursed for the teachers’ time was a good incentive. They would have liked more direct input and visits from the study team: to have had a clear timeline for study events and fewer delays, especially at the start. Parents felt the communications from the study team were good, that the questionnaire was rather long, some preferred the online version and the £10 voucher was a useful incentive.
9. The implementation of the intervention (reach, fidelity, dose, adaptations, sustainability
  The two intervention schools who completed the study had used staff meetings to discuss the PowerPoint and suggested strategies. Of the two schools who withdrew, one emailed the link to the material to all teachers in years 3, 4 and 5, and, in the other, the PowerPoint was viewed by the head teacher and SENDCo but was not distributed.
  Both intervention schools who completed the study changed the fonts and spacing in their teaching PowerPoints and worksheets, and they decluttered the classrooms. In one school, they discussed the CVI interventions at a staff meeting but did not describe using the targeted strategies with any individual children. The stories were used in all schools except one of those that withdrew. No teachers mentioned adapting any of the materials. Both intervention schools who completed the study told us they had identified children who they would like to refer for a telephone-based CVI assessment (12 from one and 2 from the other), but no referrals were actually made. Key contacts in both intervention schools described that the learning from the study (about CVI) would be carried forward in their practice. In one school, they added the PowerPoint from the intervention pack to the videos used for induction of new staff. On review of the school websites, no mention was made of the intervention.
10. Mechanisms of impact
  Teachers reported that the decluttering of classrooms was initially unpopular with children and some staff but then had a beneficial effect. One teacher commented that “they just seem a lot calmer”, and that a particular child with learning difficulties “was able to do more and more in class……which he used to struggle with”. Another teacher commented that “the biggest impact is on the SEN children”. At another intervention school, one teacher reported “it enabled them to focus on the key information”. One disadvantage of decluttering the walls was losing a previously used strategy of putting up reminders of words or spelling, for the children to use as a resource. The school staff worked around this by giving children their own booklets or signposting them to other resources, which was said to promote the children’s independence.
  Teachers commented that the study materials had made them think more about some children’s difficulties and had given them strategies to try: “it’s that digging deeper…. why have they got rubbish hand-eye co-ordination and what are we going to do?” and “as a SENDCo it has given me a greater awareness of needs….. lots of strategies that are useful across the board to suggest”. Interviewees in all schools reported that being asked for photographs made staff more aware of the appearance of their classroom displays.
  Ten teachers (3 in control schools and 7 in intervention schools) completed a CVI questionnaire (maximum possible score 50) and a self-efficacy questionnaire (maximum score 40) at baseline and follow-up. Nearly all these teachers increased their CVI knowledge scores during the study: the mean (SD) score change was 15 (3.5) in the control arm and 18.1 (17.5) in the intervention arm. By contrast for the same teachers, the mean (SD) changes in self-efficacy scores changed little: mean (SD) change was 1.3 (2.8) in the control arm and 0.6 (5.8) in the intervention arm.
11. Acceptability of the intervention
  The content of the PowerPoint was felt to be “just right” and gave the “right amount of science”. The laminated cards with advice for decluttering were popular, as were the stories for the children, although follow-on activities would have been good as well. Decluttering the rooms would have been easier during the summer holidays, but changes to the PowerPoints took little time and were described as “quick and easy”.
  We asked teachers about whether on-site vision tests would have been acceptable, and all reported they would have been preferable to the offer of a referral to the eye clinic: “it would make a huge difference to simplify it like that”.
12. Comparison between the teacher report and parent report for the 5Qs screening questionnaire
  The proportion of children for whom the responses reached the specified threshold for being at high risk of CVI (a response of “often” or “always” for 3 of the 5 questions) was 12/914 (1.3%) using teacher reports and 5/189 (2.7%) using parent reports. Of the 181 children with both parent-reported and teacher-reported 5Qs questionnaires, only 1 had a threshold score in the teacher reports, and none child had a threshold score in the parent reports.
13. Feasibility of using classroom photographs to provide objective data on visual clutter
  All schools returned photographs for each years 3–5 classroom at baseline and follow-up: 334 image files in total. We had asked for pictures of each wall, taken from the middle of the room but gave no further guidance. The photographs were in a variety of file formats and with varied composition and lighting; for example some contained large windows or had chairs on desks. We analysed the image files > 1 MB that were of the front of the classroom with a software algorithm to obtain the derived composite measure called “feature congestion (FC)” as explained previously. We had taken validation pictures at 2 m of a whiteboard with varying numbers of text documents and pictures, and the FC scores ranged from 11 to 22, whilst the FC scores for the classrooms were in lower range 1–4, reflecting the greater distance from the targets and the different picture compositions (e.g. no text). Classroom FC varied between schools and within schools between baseline and follow-up, but there was no consistent effect of the intervention (in some schools, feature congestion went down but in others up) as shown in the boxplots in Fig. 2, in which box “1” is pictures taken baseline and box “2” is pictures taken at follow-up, for each school. The large variations in image composition and quality mean it is difficult to associate any changes in the congestion matrix with the study intervention, as changes in classroom FC between baseline and follow-up could have been due to differences in image composition, such as including a window at one time point and not the other.

### Harms and adverse or unexpected events

A teacher in one of the control schools reported finding a video about CVI online, and after that, they decluttered the library and some classrooms. No harms were reported in interviews. We reported to the sponsor that as the study reopened, an error was made in the newly designed parents’ online questionnaire, whereby parents could see the names of other participating schools and children. This was reported to us by one of the head teachers within 24 h of the questionnaire going live. The link was immediately disabled, the error fixed, appropriate forms submitted to the University of Bristol Data Security officer and the sponsor and an apology email was sent to all parents. The reformatted link was them resent and parent follow-up data collected without further comments or problems.

## Discussion

In this feasibility study, we have demonstrated that a school-wide, multilevel intervention designed to share information about CVI with school staff was largely acceptable and was feasible. Amendments to the intervention (e.g. on-site vision assessments) were suggested which would be likely to further enhance acceptability. This is the first time, to our knowledge, such an approach has been tried as part of improving outcomes for children with CVI, although some evidence of the effectiveness of decluttering classrooms as a means of increasing children’s attention during, and recall after, lessons has already been reported [44]. It is important to evaluate the effectiveness of this approach, as CVI is often undiagnosed and a great many children could potentially be helped, if this approach was found to be effective in a full trial. The intervention, as well as the outcomes suggested for the planned future trial, incorporates insights from a Core Outcome Set (COS) for Paediatric CVI [45], which included “relevant adults being aware of CVI”. The comments from the teachers supported the findings of the COS as regards increasing awareness of CVI being beneficial, but a full trial is needed to explore whether the intervention is effective on a wider scale.

We were able to answer our research questions relating to the methods and collect useful information to improve the design of a future definitive trial. Although the hospital-based aspect (vision tests in the eye clinic) was not carried out, the participants reported they would have preferred this to be school based.

### Limitations

There were limitations to some aspects of this feasibility trial. The target of recruiting eight schools was not determined in a formal calculation of the number required to achieve a degree of precision in the estimation of parameters such as the ICC but by an informal judgement of the number of replications that would reassure us of the feasibility of study procedures. The COVID pandemic school closures and the extra demands on teachers at that time made it difficult to collect as much data as we had anticipated, limiting our ability to estimate follow-up rates and sensitivity of the outcome measures to the intervention. Although the teachers we interviewed reported beneficial effects relating to the children from some aspects of the intervention, e.g. decluttering, we did not have sufficient outcome data to explore whether the data supported their reports. There was evidence of contamination between study arms as some of the schools in the control group acted on materials about CVI they discovered themselves. This meant they carried out some activities recommended as part of the intervention, such as decluttering. The taking of photographs also alerted all schools to the appearance of the classrooms, which may have led to tidying and decluttering they would not otherwise have done — adding to the contamination bias. Recommendations to declutter school settings have become more widespread as part of the pandemic response and as part of a longer-term trend to adapt schools to the needs of children with neurodiversity. All these limitations may have reduced our ability to detect any changes due to the intervention. We did not include prespecified progression criteria to inform the decision about whether a future definitive trial would be feasible, although these are frequently used and are recommended. This was because we wanted to consider any proposed design changes and the qualitative data on acceptability and implantation when making this decision, and quantitative progression criteria would not help us address these [46].

### Generalisability

The baseline data are likely to be generalisable to a future definitive trial. Similarly, the data on the implementation and acceptability of the intervention are likely to generalise to other studies and provided clear guidance as to what the teachers liked and what they thought was problematic. Data from this study and a planned future trial would be most generalisable to mainstream primary schools in the UK and/or countries with similar educational practices. There may be limitations to generalisability for the proposed on-site eye tests in countries where healthcare for children is not offered in schools; however, the school-based aspects of the intervention would still be applicable.

### Interpretation

Recruitment using “cold calling” with emails and letters had a low yield, but other studies have successfully recruited primary schools into cluster RCTs [47], and a review of their recruitment methods would be helpful. The schools engaged with data collection and mean response rates with the proposed outcome measures were good (> 90%) for teacher and child questionnaires but were lower (< 40%) for parent questionnaires. Similar results for parent responses, for example 29% of parents returning follow-up resource use questionnaires, have been reported in other school-based cluster RCTs [48]. The schools (clusters) varied regarding the proportion of data returned from each participant group, and monitoring data returns from schools individually during a future trial may help avoid ascertainment bias.

The in-school aspects of the intervention were perceived as beneficial by the staff, and teachers reported mechanisms of change that were in keeping with the programme logic [20]. The request for on-site assessments rather than referrals to a clinic was useful and was similar to visits from other health professionals, as reported in the SEN-related costs questionnaires (Table 4).

### Implications for progression

We discussed the results with our steering committee who commended the team on achieving much in difficult circumstances and noted that several improvements and innovations in the trial methods were made possible with the data. They recommended disseminating the results, liaising with education policymakers and teachers’ representatives and preparing plans for a full trial to present to stakeholders, including the suggested amendments. These include using different strategies for recruitment, a clear timetable being given to schools at the outset and on-site eye assessments instead of asking the GP to make referrals to the eye clinic and a shorter questionnaire for parents with vouchers as incentives. Whilst randomisation of more schools in a full trial will achieve greater comparability between the study arms, randomisation should be stratified by important prognostic factors to ensure they are balanced. This will facilitate subgroup analyses in the full trial, to explore whether the intervention’s effectiveness varies by age, gender or socioeconomic status. Monitoring data returns from each school (cluster) separately, with a bonus for achieving a minimum threshold such as 85%, is advisable to help avoid bias due to differential ascertainment. Reducing the burden on schools by providing a researcher to help with child-report questionnaire sessions and to take the classroom photographs may reduce attrition of schools. Photographs need to be standardised regarding content and format. More guidance to the school staff regarding the use of targeted strategies may promote their use. A future study could monitor intervention fidelity using how often these targeted strategies were employed.

The contamination between arms that we observed was partly due to the pandemic but also to teachers possibly being alerted to the existence of CVI by the study materials and electing to use strategies such as decluttering if they came across them elsewhere. A future full trial could aim to reduce contamination by amending the study design and/or the participant information leaflets (PILs). For example, if the design included the control group being offered, the intervention immediately after follow-up data had been collected; on the proviso, there had been no changes in their usual practice during the study or alternatively using a “stepped wedge” design in which all clusters received the intervention but after randomly allocated delays. The PILs could be written without including the phrase “CVI” and giving far less information at the outset about the intervention. Analysis of the results would be by intention to treat, but a planned per-protocol analysis could also be conducted (if any control schools independently adopted elements of the intervention), and the results were compared to investigate the effects of possible contamination. Discussions with families of children with CVI, with schools and with methodologists would be useful to identify the most effective and acceptable ways to avoid contamination between study arms in a future trial. However, there may be future changes in educational practice that interact with the intervention, and a detailed process evaluation, with attention to variations in school context, will help to provide explanations of variations in intervention effectiveness, as recommended by the MRC guidelines for complex interventions [18]. A further benefit of a full trial with a PE would be to triangulate child-reported, teacher-reported and objective (from DfE) and health economic data on whether the effects and cost-effectiveness of this intervention were as intended and how they varied between settings, as this information would be needed before recommendations could be made for widespread use.

## Conclusions

We conclude that with the changes suggested by the data from this feasibility trial, a full-scale cluster RCT is feasible and should be presented to stakeholders for evaluation, then with their input and if appropriate, submitted in a bid for funding.

## Supplementary Information

Supplementary Material 1. Figure 1 shows the baseline logic model for the intervention and mechanism of action. Table 1 shows school characteristics relevant to the process evaluation. Table 2: Responses to brief survey questions about intervention use. Table 3: Teacher self-efficacy scale, from Schwarzer, Schmitz, & Daytner, 1999. Table 4: The CVI knowledge survey, from Pilon-Kamsteeg et.al 2019.

Supplementary Material 2. Table 2.a: Baseline assessment data from Children, Teachers and Parents, for all and by Special Education Needs (SEN) status. Supplementary table 2b: Mean (95% CI) change in questionnaire score (follow up - baseline) for participants with scores at both timepoints, by study arm and by SEN status

Supplementary Material 3. Table S3a. Frequency of parent-reported healthcare resource use at baseline and follow-up. Table S3b. Descriptive statistics of healthcare and out-of-pocket costs reported by parent/carers at baseline and follow-up. Table S3c. Descriptive statistics of parent/carer time spent supporting children in their education or health. Table S3d. Descriptive statistics of calculated child utility scores, mapped from the PedsQL to the CHU9D.

## Data Availability

Data will be available on reasonable request.

## Abbreviations

CVI: Cerebral visual impairment
SEND: Special educational needs and disability
RCT: Randomised controlled trial
