# Supplementary material for: A school-based intervention to improve mental health outcomes for children with cerebral visual impairment (CVI): feasibility cluster randomised trial
Source: Pilot Feasibility Stud. 2025 Mar 3;11:24. doi: 10.1186/s40814-025-01603-x (PMC11874832; doi:10.1186/s40814-025-01603-x)
Supplement: Supplementary file 1 — Supplementary Material 1. Figure 1 shows the baseline logic model for the intervention and mechanism of action. Table 1 shows school characteristics relevant to the process evaluation. Table 2: Responses to brief survey questions about intervention use. Table 3: Teacher self-efficacy scale, from Schwarzer, Schmitz, & Daytner, 1999. Table 4: The CVI knowledge survey, from Pilon-Kamsteeg et.al 2019. [file 40814_2025_1603_MOESM1_ESM.docx]

# CVI Project Feasibility Process Evaluation Findings

Anna Pease and Trudy Goodenough

April 2021

## Introduction

This report summarises the findings from the CVI Project Feasibility Study process evaluation. The feasibility study began in December 2019, and completed follow up data collection in March 2021, due to school closures caused by the Coronavirus pandemic. Our process evaluation aims were to explore how the intervention is implemented and provide context for the interpretation of the feasibility trial outcomes.

The process evaluation combined: (1) semi-structured qualitative interviews with (2) brief surveys, (3) documentary analysis, and (4) photographic ratings of classroom clutter. The report is divided up by each activity.

Logic Model

Figure 1 shows the baseline logic model for the intervention and mechanism of action.


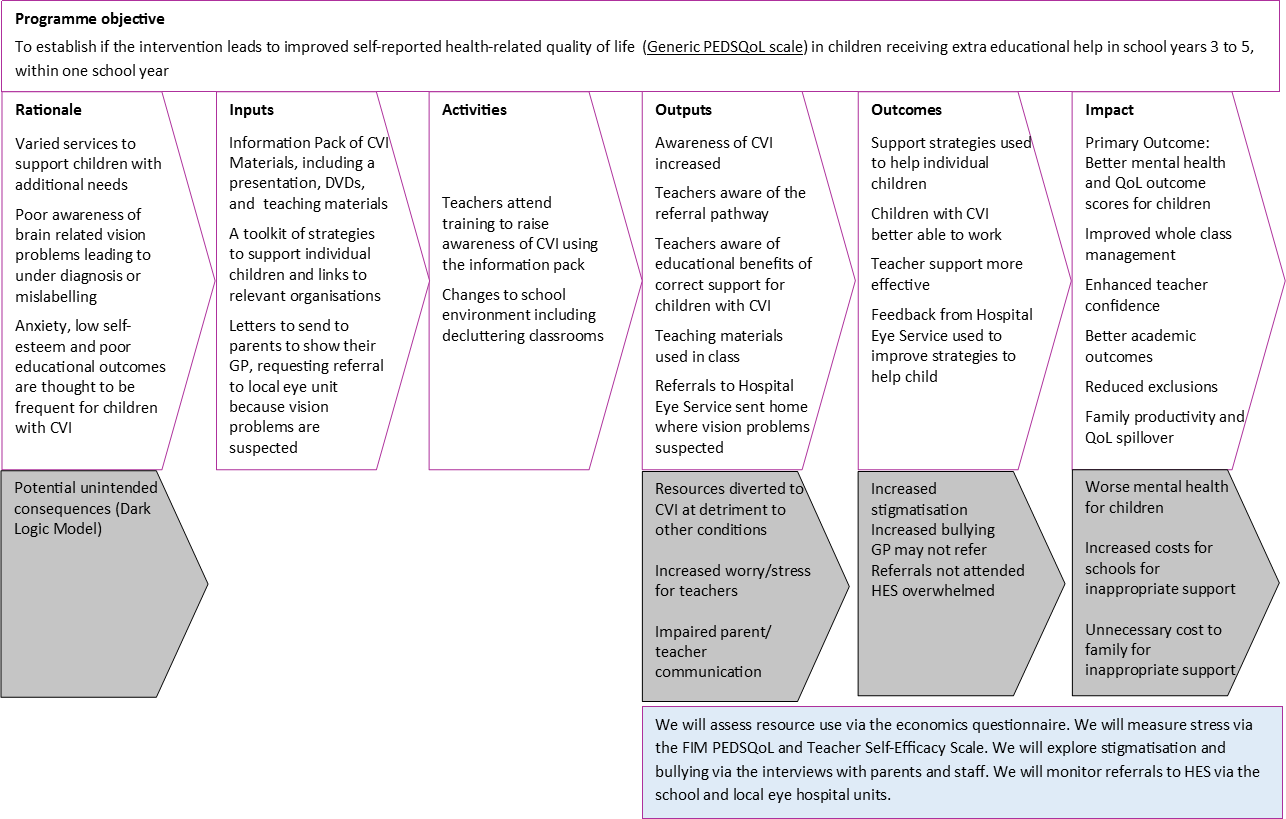


## Qualitative Interviews

- 1. ***Methods***

Four sets of semi-structured qualitative interviews were carried out:

1. Baseline interviews with school key contacts (Mar-Oct 2020)
2. Post-intervention interviews with school staff (Dec 2020 – Mar 2021)
3. Follow up interviews with school key contacts (Mar 2021)
4. Post-intervention interviews with parents of children included in the trial (Mar 2021

Further semi-structured interviews with NHS Orthoptists were originally planned but did not go ahead. These interviews would have focussed on the referral process from schools to the NHS and experiences with completing assessments for children with suspected CVIs. Since no referrals took place during the study period these interviews were not possible. We have instead incorporated views on the potential for referrals and possible onsite provision for vision assessments into the interviews with school staff and parents.

- - 1. *Participants and sampling*

For baseline and follow up key contact interviews, one person from each school who had acted as our main contact for the study was eligible to take part. Of the 7 schools enrolled at baseline, the Special Educational Needs Co-ordinator (SENCO) was our key contact in 3/7, the head teacher in 3/7 and assistant head teacher in 1/7. Table 1 shows school characteristics relevant to the process evaluation.

| ID | Arm | Number of Teachers across y3-5 (2019/2020) | Key contact |
| --- | --- | --- | --- |
| SCHOOL 1 | Control | 3 | Head Teacher |
| SCHOOL 2 | Control | 6 | SENCO |
| SCHOOL 5 | Control | 3 | Head Teacher |
| SCHOOL 6 | Intervention | 9 | Head Teacher |
| SCHOOL 3 | Intervention | 3 | SENCO |
| SCHOOL 4 | Intervention (withdrew) | 3 | Assistant Head Teacher |
| SCHOOL 7 | Intervention (withdrew) | 8 | SENCO |

The school staff interviews were carried out with those nominated by the key contacts, usually class teachers but Learning Support Assistants were also eligible.

Parents for interview were identified via the baseline and follow up trial measures – parents were asked if they would be happy to be contacted about an interview, all those that gave consent to be contacted were sent an information sheet and consent form.

Following the March 2020 school closures, two intervention schools withdrew from the study, leaving 5 schools in total, 2 intervention and 3 control.

All interviews were carried out by telephone. The topic guides were based on guidance from the Medical Research Council (MRC). [1] The seven domains to be included as topics for discussion as recommended by MRC guidance were: implementation of the intervention, mechanism of impact/change, programme differentiation and usual practice, acceptability of the intervention, sustainability, context, and trial processes. An example of the topic guide is Appendix A

- - 1. *Analysis*

Interviews were audio recorded and transcribed verbatim. Transcripts were checked for accuracy and uploaded to NVivo 12 Pro (QSR International Pty Ltd) for coding. A framework-guided analysis was applied to the transcripts, to enable rapid development of the themes relating to the broad categories of the MRC process evaluation domains.[2, 3] This approach allows for the development of initial themes, which are then conceptualised and used as a framework for coding. Four transcripts (2 key contact, 1 teacher and 1 parent) were double coded to improve robustness of the analysis. Analytical decisions were discussed with the rest of the study team and resolved in order that a consensus was reached.

- 1. ***Results***
     1. *Baseline interviews with school key contacts*

Seven interviews were carried out with one person from each school in the study. These took place following trial randomisation and so included four schools in the intervention arm and three in the control arm.

Main findings:

1. ***Implementation of the intervention***

At baseline, interviews took place shortly after schools in the intervention arm (n=4) had been sent the intervention pack (a suite of resources to raise awareness of CVI and implement universal and targeted strategies to support children who may have CVI). Their experiences with it were largely positive, and they described two phases to implementation: staff training using the PowerPoint presentation and the use of resources in the classroom. Key contacts described how staff felt that the content was new and this made it more interesting.

*“I think it was quite interesting for them, probably not something they have really considered before.” SCHOOL 3*

They spoke highly of the presentation, especially the mix of relatable content and scientific evidence.

*“…give us some science, give us some real what’s happening behind the picture of the brain looking like it’s got that real scientific thing, but not too much, […] and then at the end there you had your toolkit didn’t you that said what are we going to do about it. It had a good learning shape to it is what I would say.” SCHOOL 6*

Implementing the resources had already started in 3/4 schools, with the other describing their plans for this. The schools who had implemented some of the strategies focussed on universal measures like clearing the classrooms, changing worksheets:

*“We have done the plain backgrounds, we have got rid of the clipart bits and the little animations which has been good. I think first of all the children found them slightly more boring than what they have been used to. What I would say though is actually after a while it became better because it was just the essential information that was on there, and it enabled them to focus on the key information that we wanted them to do, and you could point them to… for example what I would do is put the mathematical definitions on the board, that’s the word, that’s what it means, and you were able to really draw their attention to that. We have also within lessons tried to restrict the amount of PowerPoint slides we use within a lesson now” SCHOOL 3*

Staff also liked the animal stories for use in class – two teachers (key contact) and (classroom teacher) commented that while they were a useful resource, they didn’t really know what to do next to follow it up (notes from call, not recorded, SCHOOL 4).

*“What I struggled to do was then to… if you linked that… I tried to link it in to why our displays are like they are, but I don’t know if that went by the by with the children. They were just like oh okay, and then the lesson just… if you know what… it just went back to normal. So it was the stories were good for that lesson in that moment, but then being able to go further than that, that was where I struggled. I tried to pigeonhole it in, so that was the only thing that I would say about that, and actually if we could have had… and I think that’s been what other teachers have found, that they were able to drop the bits and use stories, but then they weren’t able to go much further with them, so possibly in the pack some ideas of how they could be used within the lesson, or fit in a little bit more, might have been beneficial.” SCHOOL 3*

Clearing classrooms also was in line with the changes required to reduce coronavirus transmission:

*“I want to see your rooms being cleared and actually with coronavirus going on I’ve still got staff clearing rooms and getting it ready for what we’re trying to do.” SCHOOL 6*

Key contacts particularly like the laminated cards with universal and targeted strategies:

*“I thought that was really good actually because it was what I liked about it, it was very clear what they needed to do, because you had it on a plastic covered thing exactly what you were achieving. I thought that was great because it was brief and they could just pick it up and they could just see exactly what they needed to do in terms of that.” SCHOOL 7*

1. ***Mechanism of impact/change***

The impact of using the implementation was described by all intervention schools but was limited in scope due to the timing of the interview. Early indications were that the pandemic had made making additional changes very difficult, but those changes that had been made seemed to be having a positive impact so far.

*“I think with the coronavirus it’s just put a big spanner in the works because we just don’t know what’s going to be priority when we get back because we’ve got SATS coming up, so that would be a massive priority.” SCHOOL 6*

*“I think the teachers’ awareness of how their classrooms are setup, what’s displayed around the classroom, I think that’s been quite a big one. I am noticing so when I do a book look or wandering around obviously before lockdown in terms of changing the background colours of PowerPoints, printing things out, the different fonts, the different sizes, I think those are quite quick wins for the teachers that they could do without a lot of change to their practice, and those types of tweaks to their high quality teaching I would say would be attributed to your project.” SCHOOL 3*

1. ***Programme differentiation and usual practice***

One school in the control group described their pre-existing awareness of the visual environment:

*“I would say the school we are very aware of the visual impact of space, and it’s something that we talk a lot about every now and then, because we’re very keen that we don’t overstimulate if possible from a visual point of view. So we do talk a lot about colour and things like if you’re backing a board trying to use neutral colours and to be aware of things like that. So I think as a school we do take note of that anyway.” SCHOOL 2*

Another of the control schools described how they had changed the visual environment in the school.

*“So I don’t know if that’s a link to you but we took that decision, and in fact I think from September we’re going to go hessian backing and actually get rid of colours, so I don’t even know if that’s even linked, but that’s interesting that we are ourselves moving.*

**(Interviewer) Why did you make that decision?**

*Just because I just felt that… I actually made it in the library really, for our library, I felt that our library was too busy and too bright, so I took the decision for the library and then actually we just did it everywhere.” SCHOOL 1*

1. ***Acceptability of the intervention***

All schools spoke positively about the organisation of the intervention pack and resources, but some struggled with decluttering classrooms, mostly due to teachers’ usual practice of displaying their students’ work.

*“The one that’s caused the most controversy has been taking things down off the walls, that’s quite interesting the staff’s reaction to that. They are very hesitant to do it, they are complaining that their rooms look bare, they are worried.” SCHOOL 6*

The other intervention school suggested making intervention resources available during the summer holidays so that staff have time to implement the decluttering before spending time making their usual displays.

*“then we would have said we are doing this fantastic project, we need you to design your classrooms like this, and that would have given them… because a lot of them obviously take thought on their displays, where I’ve gone look we need to change them they are like… it takes them so long to do it.” SCHOOL 7*

In terms of referrals, schools preferred onsite assessments from orthoptists, if these were available. They stated reasons including easier logistically, but also less likely to worry parents as they could reassure them that it was nothing to worry about.

*“So one of the difficulties we have with some of our parents is their cognitive ability and also how they feel around other professionals, they are often quite intimidated by professionals, and so myself and the pastoral lead do accompany to various meetings with parents, just to ensure really that they go because quite often they will cancel for various reasons, speech and language, things like that. So I think if it could be done in school where the school has built up the trust with the parents and it could be done in conjunction I think that would be brilliant.” SCHOOL 3*

1. ***Sustainability***

Only one intervention school mentioned sustainability at baseline, suggesting that they would incorporate the strategies into standard procedures for supporting students who may be at risk of having CVI.

*“when it comes up in conferencing talking about those particular children with educational behaviour problems is there anything that we can use from this project that might actually support what’s going on with that child.” SCHOOL 6*

1. ***Context***

The coronavirus pandemic did come up for those interviews which took place after lockdown (March 2020). One intervention school hoped that the project would have longer to run as a result of the pandemic.

*“I think we need longer, I feel like the project is behind its timescales now, probably corona has just absolutely kyboshed those, so I don’t know whether the project is going to run slightly longer in terms of going over into the new academic year.” SCHOOL 6*

schools also refereed to other approaches taken within the school in response to the needs of the school population or in conjunction with other research studies that they were involved in during the CVI Project, as seen in this example from one of the control schools.

*“Before lockdown, so not last September, the September before, we worked… we removed a lot of the fuss. We stripped back our classroom, so looking at what’s good for all children and children with sensory issues really struggle with such big classrooms with everything everywhere, and so our classrooms are already stripped back so that there isn’t that sensory overload. Things are on display boards, but not everything crammed everywhere half falling off, things are put up that are purposeful. SCHOOL 5*

1. ***Trial processes***

All schools (n=7) discussed the process of engaging with the study measures and appreciated the study team’s efforts to organise and communicate clearly and comprehensively.

*“the fact that they had their own box, they had even provided us with staplers if we needed to use them and things like that. So it was easy to administer from our point of view because it was well organised.” SCHOOL 2*

Two schools, one control and one intervention, commented on the study timing:

*“The other thing I would feedback is that I think we need longer, I feel like the project is behind its timescales now, probably corona has just absolutely kyboshed those, so I don’t know whether the project is going to run slightly longer in terms of going over into the new academic year, but I feel that we were waiting for things to come and just it was almost a term really.” SCHOOL 6*

*“I suppose because at the time when it initially started we were like yes let’s commit to this as one of our focus areas for this year, and actually by the time it got going we might have been moving on to something else if we hadn’t got going if you see what I mean? So from that point of view I would say it was a little frustrating. Just because it hasn’t got momentum at the start, we then were not that we forgot about you, but had our time been taken up with you we wouldn’t then maybe have done some of the other things. But we then started doing other things and then it was like oh goodness, don’t forget about that.” SCHOOL 1*

But schools also recognised that some of the length of the study ‘set up’ period was due to their slow response to CVI Project requests for information and data, including classroom photographs.

One school wondered whether enrolling only one class would have been easier:

*“In terms of seeing trends in any of our data I think as a small school one class… maybe it would have been easier to manage.” SCHOOL 5*

Other schools stated that money was a factor in signing up: *“like I said the reason I was motivated to do it was because you said that there was money available” SCHOOL 6*

One school was confused about what the intervention would entail and had expected screening to identify children with CVI prior to a support package being given (notes, not recorded, SCHOOL 4).

Anonymity of child self-report questionnaires was maintained by most schools, with one suggesting that we provide additional definitions for some of the questions in order to help them to complete it*. “so in some ways it would be useful if there was a definition for teachers to be able to readout so that you guys get the same information back” SCHOOL 6*

However, anonymity was not maintained at one intervention school: *“it was interesting seeing some of the responses on some of them. We’ve got some children with some needs and difficulties at home, and it was interesting reading how they responded with their self-esteem and how they felt that they couldn’t… we’ve got one family that she’s got triplets but her children overeat, and it was interesting to see how they had filled out their forms, they were very honest and you realised how much it was having an impact on them and their mental wellbeing. So some of the responses I think, some of the teachers were interested with some of the responses.” SCHOOL 7*

- - 1. *Post-intervention interviews with school staff*

Two intervention schools had withdrawn by the time staff interviews were conducted (SCHOOL 4 and SCHOOL 7). Nine semi-structured interviews with school staff were conducted, from 4 schools (2 intervention and 2 control). Seven were with class teachers, two with learning support assistants and one with a learning support assistant who was also a qualified teacher.

- 1. ***Implementation of the intervention***

Staff in the 2 intervention schools described how they had used the resources to declutter both their classrooms, make worksheets simpler, increase font sizes and use more easy to read fonts.

*“she went through what was in the packs and our expectations as what she wanted is to do with regards to the project. So for example keeping everything within the board space, not having things above the board which… not having things on the windows, font sizes need to be bigger” SCHOOL 6*

There was some confusion over where font choices had come from, whether it was the CVI Project intervention or one of the linked resources.

*“I know that [name] has said that we will be using the two recommended fonts and the size on worksheets and letters and keeping the slides and everything the information to a minimum and not putting loads of stuff on it.” SCHOOL 6*

- 1. ***Mechanism of impact/change***

Decluttering of classroom walls was controversial, and teachers could see both benefits and potential problems, especially for children who were used to having key concepts on walls and windows to support their learning.

*“So I will have a little display saying what all these technical words are, but obviously where I can’t have a display board anymore it’s me constantly telling them what it is. So yes and no, it has helped, but then for some of the ones that can’t retain what a simple past, present, progressive is, sometimes it’s nice to have that up so they can go, “What’s that? Oh yeah that’s that.” So then they can join with the rest of the lesson, otherwise sometimes they sit there going, “I don’t know what that is, don’t pick me, don’t pick me because I don’t know what that it,” and then that’s when you see the behaviour change because then they’re anxious and they’re worried. So swings both ways.” SCHOOL 6*

But another teacher described how this was ‘worked around;’ in her classroom and the benefits that the changes had made:

*Because we would normally have all our word banks up on display my team has now got we’re trying out a vocabulary toolbox, so all the children have their individual books, and all the word banks that we introduce for each of the subjects across a unit of work they have them in their toolbox. So they’re referring to their toolbox which is an exercise book, and they’re finding the correct toolbox which is meaning they are not having to look around the room or even at the board for it, they have got it in front of them so there’s less looking around, so there’s less business. I think also a development of being more individually organised because they have got the resource they need in front of them, and it’s making them responsible for having that stuck in their book. SCHOOL 6*

Another teacher described the calming effect of decluttering.

**“(Interviewer) Have you noticed a difference within the class since you have done that?**

*Massively. The first day the kids came in they didn’t like it at all initially, because they are used to colour all around them. So I would say that the class feels less busy visually. Initially the children’s opinion of it was really negative, because they were used to having stuff everywhere, and then I would say as they have got used to it there is a, this feels really strong to say it’s calmer, how do you measure that calm? […] isn’t suddenly going to have a bright set of words hanging in front of their face. So I would say that has been a lot calmer. I would say the biggest impact is on the SEN children.” SCHOOL 6*

Seeing the benefits for individual children helped staff to go along with decluttering. They reported children being calmer and more able to focus on tasks due to fewer visual distractions.

*“I think that children they just seem a lot calmer. I remember when that child that I told you about at the beginning who had the quite severe learning difficulties, he was amazed by the change to the classroom, but very quickly I found that he was able to work in our classroom more when… so he often had to be taken out because he couldn’t cope with the noise, the visual stimulus of the classroom, and so he would be taken out to work by his one to one, because his behaviour would start kicking off so he couldn’t be in class anyway. But then he did start to calm down I feel, and he was able to do more and more in class including things like listening to a story which he couldn’t always do, having our morning time in there which he used to struggle with. It wasn’t… he couldn’t spend the whole day in there still, but I think that’s for a lot of reasons, but I genuinely feel that the change in classroom displays did help him.” SCHOOL 6*

- 1. ***Programme differentiation and usual practice***

The staff from schools in the control group reported increased awareness of classroom displays from the act of taking the photographs as part of study data collection.

*“So I would say it’s made us more aware. I would say just from the fact that a teacher was coming around to take photos of our classroom displays etc, it made us all really look and focus more on our displays and what actually they look like, taking a picture.” SCHOOL 1*

Additionally, the introduction of the CVI Project into control schools raised awareness of CVI and visual difficulties that might be relevant for children within the class:

*“I know that we saw the little video online about it, so that opened our eyes what it was all really about.*

**(Interviewer) So when you say video online do you know where that had come from?**

*I think it had come… had it come from your CVI Project, but then had a link somewhere else?......So when it came out and had a little look I didn’t really know before we started the project what CVI was about so I did take myself off just to have a look at some other websites about CVI. So the NHS website I looked at I think, and probably is there a CVI awareness website as well?*

**(Interviewer) What sort of things were useful for you from those?**

*Just about the sorts of things that I was looking out for in my class really a little bit more, about sorts of things that I need to be aware of in order to know what CVI displayed, what symptoms they displayed*.” SCHOOL 1

- 1. ***Acceptability of the intervention***

Again, this was mostly tied up with whether or not decluttering classroom wall displays was acceptable. Time taken to make the changes also indicated that staff prefer interventions that makes sense and don’t take very long to implement.

*“it took an hour or so to take things down, and then an adaptation of resources, but you were going to make those resources anyway so you just made them in a slightly different way. So I wouldn’t say that a lot of time has been invested in the preparation of making your classroom look a different way.” SCHOOL 6*

They also expressed an interest in members of the study team visiting schools to explain how it would work.

*“I do think it would have been good to have someone who came and talked about what you were trying to achieve, so at the beginning I think that would have given it more status. I think some schools maybe wouldn’t want to dedicate the staff meeting time to it, but actually if someone came in initially and did the talk, and then said I am going to come back and be able to say even if you’re not one of the chosen schools this is what we found out, maybe you want to try some of these things, actually then it’s worth being part of that study. Or here’s a look at the resources that you didn’t get but you could… we found really effective. That would be a good way forward.” SCHOOL 3*

- 1. ***Sustainability***

Staff described how the changes to classrooms might only be temporary, and how factoring in changes to staff inductions will be necessary to keep everyone on board with the changes long term.

*“I think we could face is that we’ve got a lot of staff who will be aware of the CVI project and are used to how the classroom works and how it looks, and how our PowerPoints work. It’s just then getting new staff trained up and aware of the project so that it keeps the ball rolling forward.” SCHOOL 3*

- 1. ***Context***

school closures and new virus control procedures in schools made everything more complicated. No referrals were sent to the eye service, despite plenty of willing.

*“we were definitely very keen as teachers to put forward as many children for that kind of thing as possible, because we want to do the best for them and help them.” SCHOOL 6*

- 1. ***Trial processes***

Staff appreciated being given time to fill out questionnaires and found it didn’t take as long as they expected. They were particularly interested in hearing about the findings of the study. Some felt that communication from senior staff could have been better.

*“I don’t think the communication from our seniors was that great about the project.” SCHOOL 6*

They also felt that more visits from the study team might have helped motivate staff and keep things moving in the school.

*“We did make it work as a staff team, but also I think on occasions for the rest of the staff it might be that oh no the head and the deputy head and the senior leadership are giving another thing to do, and they didn’t see it as an external project that we were going to test and look at, and the reasons why we were going to be doing it, and actually it can be quite good on occasions to have external people come in and say we’re going to try this because of this, it’s not something that the leadership team and the staff tend to go oh no not them giving us something else. So in that sense it might have been good to have somebody else come in to do it.” SCHOOL 5*

The timing of the initial introduction to the study and the confirmation of which arm the school would be in was also raised:

- - 1. *Follow up interviews with school key contacts.*

Four follow up interviews were completed with our key contacts in person, with one by email correspondence as the interviewee was unbale to speak with us.

1. ***Implementation of the intervention***

In one school, all resources were used, rolled out across the whole school not just year groups in the study, for continuity. Staff meetings to disseminate, in two parts – universal then targeted. They have taken decluttering to mean classroom walls but also learning materials. Wanted to use referral process more.

*“So about October/beginning of November, over that half term period we were looking again at are there children who are showing potential signs that we need to look at this CVI, and then we hit back into December and the second lockdown and then January, and we haven’t managed to pick those back up again.” SCHOOL 6*

And a more detailed example of how the intervention was implemented:

*“So for example if you were looking at a maths sum, let’s do it nice and simply, you were looking at some column addition for example, instead of doing one column addition and then working it through with the children on the board, and then looking at a different column, a different set of numbers, we have started now to rewind the board, undo what we have done, and rework exactly the same model which we think is having the impact on any children who may have a CVI that they can focus on that and then have a chance to see it all through again. So if it has been difficult they can actually get a second chance at it if that makes sense?” SCHOOL 6*

*“The intervention pack was built into our afternoon sessions and generally fitted into science or phse.” SCHOOL 3*

And a more detailed example of how the intervention was implemented:

*“So for example if you were looking at a maths sum, let’s do it nice and simply, you were looking at some column addition for example, instead of doing one column addition and then working it through with the children on the board, and then looking at a different column, a different set of numbers, we have started now to rewind the board, undo what we have done, and rework exactly the same model which we think is having the impact on any children who may have a CVI that they can focus on that and then have a chance to see it all through again. So if it has been difficult they can actually get a second chance at it if that makes sense?” SCHOOL 6*

schools definitely wanted onsite assessments for CVI. They felt parents would be reluctant to visit a hospital currently and if it was onsite it would take out the complexity of the GP referral.

*“So absolutely, if we could have some kind of a screening here that would be phenomenal, and I think you would certainly get a day’s worth of children that we would be able to put through books. It would make a huge difference to simplify it like that.” SCHOOL 6*

1. ***Mechanism of impact/change***

Both intervention schools key contacts described how it had changed their thinking, encouraging them to look for reasons behind differences seen in children and that this was a positive change.

*“Actually I suppose that’s it, it’s that digging deeper, that’s not an okay answer they have got rubbish hand-eye coordination, well why have they got rubbish hand-eye coordination, and what are we going to do? And if it’s a CVI then there’s a reason there so what are we going to do about that, if it isn’t what are you going to do to help them develop the hand-eye coordination, and I think it’s that stopping and thinking, and that’s what the video made me do, stop and think about children in the past that I had gone oh yeah you’ve just got rubbish hand-eye coordination, and I’ve moved on too quickly and never thought why.” SCHOOL 6*

*“As a SENDCo it has given me a greater awareness of needs and when someone comes to me with concerns about a child , I now consider whether CVI needs to be considered. The information provided also gives me lots of strategies that are useful across the board to suggest.” SCHOOL 3*

1. ***Programme differentiation and usual practice***

The pandemic meant that control schools were also changing their classroom environments, to control virus transmission. Much more outside time and no assemblies.

*“Yes, undoubtedly, not least because of Covid, there are things that are slightly restrictive in terms of movement, resources, and in the way a school operates too, because you keep children together and just their bubble, whereas you would have had some interactions across those groups of children previously.*

***(interviewer) Have you made any differences to the structure or the layout of the classrooms?***

*Not massively different other than you have to have children facing forwards, so that’s the same for everybody, every child in every school, whereas you might have sat in groups. But I think quite commonly you would have had a slightly mixed approach in the classroom where you would have some doing that and some with group tables prior to that.*

***(Interviewer) Any in the equipment that you would have had around?***

*Yeah, I would have had more things out, so trying to keep to the minimum what is out. However if it’s equipment that’s needed for learning it would come out and go away again rather than perhaps just be there, for example if I’m doing maths and I need some bean counters I would put them out and put them away again, whereas prior to that I might have kept them out, that’s just a small example.” SCHOOL 2*

1. ***Acceptability of the intervention***

In one school, the intervention was rolled out across the whole school and was acceptable.

*“I know the original thing was for year three to five but we actually rolled it out across the whole school in order to have that continuity and that sort of a professional development for everybody if that makes sense?” SCHOOL 6*

The second intervention school noted that:

*“The packs were easy to use as standalone sessions or to slot into the scheme of work. The packs contained a lot of information and children loved the stories that accompanied the intervention packs.” SCHOOL 3*

1. ***Sustainability***

One intervention school described how they will change policies to include consideration of the visual environment, embed the resources into staff inductions and use the universal measures in SEND policies.

*“as a result of like I say the CVI and the research on working memory and everything else that’s going around in the school we are changing our expectations around the school in terms of displays and how worksheets are formatted, and also that expectation of don’t just gloss over things with a child, don’t just shrug and go oh well yes. So those are the three main things in policy change, it will be what classrooms should look like, […] and using the phrase universal measures if I’m honest, measures that we do for our SEND, and that’s a policy change that we’re bringing in. Also what’s coming in as part of it we have a bank of videos that any new members of teaching staff this is, where they watch a video about autism, […] and the CVI one is in amongst that list of videos that new staff need to look at.” SCHOOL 6*

The second intervention school key contact noted that staff have changed their everyday approach due to some of things they had learned from the intervention including: “*How children may present and what to look for; a raised awareness of  how “common” issues can be; how to refer on if needed and considering the use of font and spacing.” SCHOOL 3*

1. ***Context***

The pandemic definitely impacted on the intervention and trial processes.

*“It was at times only I think because of the sheer length that this all went on for with Covid personally I found it difficult to go now where did I go to find that but of information, and like I said that’s probably more because March came and your brain gets swept with one thing, and then you’re reopening and everything else, and then you’re coming back to something, so it’s not solely the CVI thing that’s been challenging to find where you were. So it was useful, so we’ve been backwards and forwards to them, we really have, and we have shared them in different ways. I would have liked to have got time to share it with my LSAs as well, but we just haven’t got there at all.” SCHOOL 6*

In one intervention school they did not get to make any successful referrals to the eye service but would still like to pursue this as an option.

*“So about October/beginning of November, over that half term period we were looking again at are there children who are showing potential signs that we need to look at this CVI, and then we hit back into December and the second lockdown and then January, and we haven’t managed to pick those back up again.” SCHOOL 6*

1. ***Trial processes***

One intervention school would have liked a timetable of when things needed to be done by to manage expectations and timeframes.

*“thinking about the children and everything else, have a timeline for how you want it run, that you share with the leadership. I imagine that it works better in schools where you’ve actually had a leader on-board, and actually to know the big picture of where you were heading, and the whole calendar would have meant that I think it would have been easier to fit that in with what we were doing as a school, and we would have probably been able to alert you if we were falling off trajectory quicker, and equally you would be able to alert us if that made sense if we were falling off.” SCHOOL 6*

Most schools were still happy with the processes at follow up and appreciated the thought put in to the data collection processes.

*“The process of it was fine. Doing the survey in the classroom actually that baseline one I did with the class as well, it was very easy, we didn’t need to use any of the adapted questions at all with our children in our particular mainstream setting, so it was easy for children to understand, it was easy to administer and to bring back together. You could see that it was highly organised with all your boxes, you had thought through how this could all work.” SCHOOL 6*

The control schools also would have liked a team member to have visited.

*“because obviously you can give me information via your leaflets and telephone calls, and then I have to share that with the staff. So sometimes it might be better if it was straight from the horse’s mouth in terms of promoting that project possibly.” SCHOOL 2*

*“I was relaying that information third hand really. I think it would have been beneficial, I think we might have had more collaboration, more support from staff to do it if they… I think maybe me trying to explain it… I think that’s actually a good idea to have someone come in.” SCHOOL 1*

schools appreciated the financial incentive.

*“well with schools’ tight budget that’s worth us doing that if there’s a financial incentive for us to do it. So I can’t deny that the financial incentive is quite important for schools I think.” SCHOOL 2*

They described how the children’s questionnaires prompted some discussion, especially about sleep.

One control school mentioned that fewer parents may have completed the online questionnaire compared with the paper one.

- - 1. *Post-intervention interviews with parents of children included in the trial*

Parent interviews were designed to focussed on experiences with trial processes; any changes within schools that parents had noticed including classroom organisation, changes to SEND provision; referral processes to HES in intervention schools; and their experiences of changes during the study period due to the COVID pandemic.

Sixteen parents took part in interviews. Three interviews were with parents who have a work connection to one intervention school: 2 LSAs who were also parents of children in the study, and 1 with a father who was the partner of a teacher at the one of the intervention schools. Of the 16 interviews completed, 11 were with parents from one of the intervention schools, 5 parents were interviewed covering all 3 control schools. In the intervention schools, 6 parents reported that their child had additional educational or emotional health needs, in the control schools this was reported by 3 parents. Parents reported that their children were more likely to be in school during the second lockdown (8 children in school in the second lockdown period only) than the first (3 children who attended in both lockdown periods) with 5 children not attending school in either lockdown.

1. ***Implementation of the intervention***

We asked parents to describe any changes that had occurred at their schools during the study period for any reason. We were aware from information on school websites that the classrooms had been rearranged so that all of the children sat in rows and faced the front of the class rather than in group tables. Parents reported that this as the main change in the school environment, but that for may they were now not allowed into school, thus they had little further information or observations to share.

*“No, so we don’t go into school at all which I guess is a challenge, and also because Covid changed quite a lot of the school layout, so they are all facing forward on desks now in rows and they have to stay in the same seats, and the teacher has to stay at the front in her box. So I don’t know if my children would have said anything to me, because they wouldn’t have necessarily known that it wasn’t just related to Covid. Does that make sense? Everything at the moment that changes they just think it’s because of Covid even if it’s not even remotely related”. CR Parent*

*“Well, that was a hard one, because of Covid we’re not allowed any visitors into school. If we have any worries, we have to email teachers and things. We cut down on all the face-to-face interactions, so we wouldn’t have been in the school to notice any difference.” HR Parent*

1. ***Acceptability of the intervention***

They would be fine with onsite vision assessments.

*“I think that’s a good idea if that’s something that’s possible. I can’t imagine any parent would be upset or annoyed or whatever for the school saying we think perhaps you should go to the doctor or whatever, because surely everyone accepts that the school are going to pick up on things that maybe you don’t pick up on at home.” KM Parent*

Another parent commented on the costs of attending hospital appointments in terms of time off work, travel and parking and felt that therefore onsite assessments would be beneficial to parents and children.

1. ***Context***

Parents talked about covid school closures and changes to pick up/drop off times, they noticed more covid related changes that study changes.

*“I can’t say I have noticed, but bearing in mind that we are not allowed to go in, there is nothing you can visually see, if that makes sense to you? We can’t go in school, there is nothing much visually outside. The only thing that I know is that they did that one-way system due to Covid which was working quite well.” IC Parent*

*“No, so we don’t go into school at all which I guess is a challenge, and also because Covid changed quite a lot of the school layout, so they are all facing forward on desks now in rows and they have to stay in the same seats, and the teacher has to stay at the front in her box. So I don’t know if my children would have said anything to me, because they wouldn’t have necessarily known that it wasn’t just related to Covid. Does that make sense? Everything at the moment that changes they just think it’s because of Covid even if it’s not even remotely related.” CR Parent*

1. ***Trial processes***

Parents reported that the trial processes were generally acceptable, communication with the study team was good, although interrupted by Covid. As the study lasted longer than initially planned, parents would have appreciated some ‘interim’ information from the study team.

Parents reflected on paper and online data collection with most preferring online data entry. They reported that it was more convenient and less likely to get lost on the way home and back to school.

*“I actually preferred the online……It was fast, it went straight to you, it didn’t take long, it was easy to read, you could go back, it’s just it was easy-peasy. It was easier than bringing it home, filling it in, making sure that the child gave it in the next day, there’s more ownership as a parent rather than trusting, well I do trust them, but relying on the child giving it in. The amount of times that we have had, “I didn’t realise that letter was in the bottom of my bag,” and it does happen.” HR parent*

The content of the questionnaires was deemed acceptable. Some parents felt that the questions did not apply at all to their child, with other commenting on the way that the questionnaire was set out.

*“They were very fair questions. Sometimes I would have thought it would have been nice to explain theirs, but there wasn’t much chance for an explanation, if you know what I mean? But no, yeah they were very easy to understand, easy to answer.” HR Parent*

*“I wasn’t sure whether it was the right survey sort of thing, because it was very focused on has your child… each question you answered has your child got a hearing problem for example, and then the next question would still relate to hearing even though you’ve ticked no.*

**(Interviewer) so from the practical point of the questionnaire you were ticking no and you were still getting the questions?**

*Yeah, so it was like normally on say a paper one you might have it would say if no go to question 28 or something, but you were going through ticking how often did I see a specialist, well not at all because I just told you that..” KA Parent*

The parent’s online questionnaire contained a section for any child not at school for the school-based data collection to complete. Parents described how they helped their children interpret these child completion questions.

*“He’s very keen to get help, so he is always keen to let people know when things are good and when things are bad I think. So when he’s asked his opinion I think he quite enjoys that, he likes to be able to say how it is for him, and he likes it the rating scales. We did the one with more choices I think, and he likes that. He doesn’t like having to talk too much, he likes to just say I am here, I think that works well for him.” CR Parent*

*“She was very honest, which was quite nice actually, because I will always ask them the questions, but if I don’t quite believe that they would say I would change it afterwards. But she was very honest, and she does understand her struggles, which was quite nice, because then we were working together to answer it, if that makes sense? She didn’t feel like I was undermining her, it was her answers, she could see I was putting her answers, and that was quite nice.” HR Parent*

The voucher was felt to be beneficial to encourage some people to take part.

*“But if there is that £10 you will be like oh you know what I will do it. So it might be just that tipping point.” IC Parent*

*“It felt quite long, but the Amazon voucher incentive helped.*

**(Interviewer) Did you think it was too long?**

*I don’t know. I think maybe without the incentive… I don’t work so I do have quite a bit of time, but I imagine without that incentive I would have… some people might have got to page, I don’t know, four or five or whatever and thought do you know what this is going on too long, I am stopping now.” KM Parent*

##

## Surveys

Three surveys were completed as part of the process evaluation: brief survey questions by each key contact on intervention use, the teacher self-efficacy scale [4], and a 5-item CVI knowledge questionnaire [5].

## Brief Survey Questions

Six questions were asked at the beginning of each key contact interview to gather information about how the intervention pack was used in each intervention school. Responses are tabulated below.

Table 2: Responses to brief survey questions about intervention use

| **Question** | **Response** | | | |
| --- | --- | --- | --- | --- |
| **Intervention school** | A (SCHOOL 6) | B (SCHOOL 3) | C (SCHOOL 7 - withdrawn) | D (SCHOOL 4 - withdrawn) |
| 1. **How was the training delivered?** | Staff meetings using PowerPoint, videos and discussion of how to implement the ‘universal measures. | SENDCo talked through the PowerPoint presentation at a staff meeting | Via online link and email to class teachers in years 3, 4 and 5 | Assistant head and SENDCo watched PowerPoint presentation |
| 1. **Who delivered the training?** | Head Teacher | SENDCo | SENDCo | Only watched by Assistant head and SENDCo |
| 1. **Which components of the intervention pack were used?** | Universal measures; decluttering classrooms. Restricting displays to within display boards.  Increases in font size and changes in font for classroom presentations and worksheets | Cassandra’s stories within science and PHSE sessions.  Changes to PowerPoint presentations and to homework worksheets for Maths and English. Larger font and greater spacing between lines and maths problems. | SENDCo reported that teachers would use stories and de-clutter classrooms, and perhaps after Easter 2020 trial some of the suggestions/interventions.  SENDCo had also looked at Cassandra’s stories with a view to using them but withdrew from study before this could happen. | Assistant head used Cassandra’s stories in class |
| 1. **Who received the training? How many people in each group and in total?** | Teaching staff, in years 3 to 6, number not specified. Not LSAs although head notes, she would like to, but did not have the time. | Staff at staff meeting | 8 class teachers. in years 3, 4 and 5 | Not disseminated to wider school staff |
| 1. **How many referral letters sent out to parents if any so far?** | None, but a number of children identified who would benefit from a referral | None, but 2 children identified as possibly benefitting, to be discussed first with their parents before referral formally made | None, school withdrew | None, school withdrew |
| 1. **How many students/children identified as would benefit from a referral so far?** | 10-12 | 2 | None recorded school withdrew | None, school withdrew |

## Teacher self-efficacy

The ten-item scale[4] was sent out to all teachers in seven schools at baseline, and emails sent to the teachers in five schools who had previously completed the measure in continuing schools, at follow up.

At baseline, 32 teachers completed the survey – 9 from 2 control schools (SCHOOL 1, SCHOOL 2) and 23 from 4 intervention schools. One control school (school 5) did not complete any teacher questionnaires.

At follow up, 10 teachers completed the survey, 3 from 2 control schools (SCHOOL 1, SCHOOL 2) and 7 from 2 intervention schools (SCHOOL 6 and SCHOOL 3). Two intervention schools had withdrawn at follow up, and again, one control school (school 5) did not complete any teacher surveys.

In total, there were 35 classes across years 3-5 enrolled in the study at baseline from all seven participating schools. Assuming each teacher completing a survey is from one class, we had a 91.4% response rate at baseline (100% from six schools, 0% from one), and a 41.6% response rate at follow up (as two schools withdrew, the denominator using number of classes becomes 24).

The items on the scale are shown in Table 3. The 4 point response scale comprised: Not at all true, barely true, moderately true, and exactly true.

Table 3: Teacher self-efficacy scale, from Schwarzer, Schmitz, & Daytner, 1999

| 1. **I am convinced that I am able to teach successfully all relevant subject content to even the most difﬁcult students.** |
| --- |
| 1. **I know that I can maintain a positive relationship with parents, even when tensions arise.** |
| 1. **When I try really hard, I am able to reach even the most difﬁcult students.** |
| 1. **I am convinced that, as time goes by, I will continue to become more and more capable of helping to address my students’ needs.** |
| 1. **Even if I am disrupted while teaching, I am conﬁdent that I can maintain my composure and continue to teach well.** |
| 1. **I am conﬁdent in my ability to be responsive to my students’ needs, even if I am having a bad day.** |
| 1. **If I try hard enough, I know that I can exert a positive inﬂuence on both the personal and academic development of my students.** |
| 1. **I am convinced that I can develop creative ways to cope with system constraints (such as budget cuts and other administrative problems) and continue to teach well.** |
| 1. **I know that I can motivate my students to participate in innovative projects.** |
| 1. **I know that I can carry out innovative projects, even when I am opposed by sceptical colleagues.** |

Scores for each participant were out of 40, with total scores from each question out of 4. Scores for all 32 teachers at baseline ranged from 2-4 with an overall average score of 34.6/40. Scores from all 10 teachers at follow up ranged from 2-4 with an overall average of 36.4/40.

Average scores for all teachers who completed the survey is shown in figure 2 by arm (with standard error bars).

Average self-efficacy scores out of 4 for each item for all teachers regardless of study arm is shown in figure 3:

Average self-efficacy scores out of 4 for each item for all teachers by study arm, is shown in figure 4:

Average self-efficacy scores for each item for the 10 teachers who completed both baseline and follow up surveys is shown in figure 5:

## Teacher CVI Knowledge

The five item scale was sent out to all teachers in seven schools at baseline, and emails sent to the teachers who had previously completed the measure in five schools who were still in the study at follow up.

At baseline, 32 teachers completed the survey – 9 from 2 control schools (SCHOOL 1, SCHOOL 2) and 23 from 4 intervention schools. One control school (school 5) did not complete any teacher questionnaires.

At follow up, 10 teachers completed the survey, 3 from 2 control schools (SCHOOL 1, SCHOOL 2) and 7 from 2 intervention schools (SCHOOL 6 and SCHOOL 3). Two intervention schools had withdrawn at follow up, and again, one control school (school 5) did not complete any teacher surveys.

In total, there were 35 classes across years 3-5 enrolled in the study from all participating schools. Assuming each teacher completing a survey is from one class, we had a 91.4% response rate at baseline (100% from six schools, 0% from one), and a 28.6% response rate at follow up.

The five item scale has been used in other evaluations of CVI based interventions for professionals, uses a 10-point Likert type scale as shown in Table 3 (adapted from [5])

Table 4: The CVI knowledge survey, from Pilon-Kamsteeg et.al 2019.

|  | 0 | 1 | 2 | 3 | 4 | 5 | 6 | 7 | 8 | 9 | 10 | |
| --- | --- | --- | --- | --- | --- | --- | --- | --- | --- | --- | --- | --- |
| 1. I know everything about CVI | Not at all |  |  |  |  |  |  |  |  |  | | Very much so |
| 2. I understand how complicated the cerebral visual system is | Not at all |  |  |  |  |  |  |  |  |  | | Very much so |
| 3. I understand the impact of CVI in daily life | Not at all |  |  |  |  |  |  |  |  |  | | Very much so |
| 4. I can explain the impact of CVI in daily life to others. | Not at all |  |  |  |  |  |  |  |  |  | | Very much so |
| 5. I know how to adjust the environment of a child with CVI | Not at all |  |  |  |  |  |  |  |  |  | | Very much so |

Scores for each participant were out of 50, with total scores from each question out of 10. Scores for all 32 teachers at baseline ranged from 0-7, scores from all 10 teachers at follow up ranged from 1-10.

Average scores for all teachers who completed the survey is shown in figure 6 by arm (with standard error bars).

Average CVI knowledge scores out of 10 for each item for all teachers is shown in figure 7:

Average CVI knowledge scores for each item for the 10 teachers who completed both baseline and follow up surveys is shown in figure 8:

## Documentary Analysis

We investigated whether there were any changes in school SEND documentation over the course of the study that might indicate contamination between intervention and study schools, or if there was evidence of control schools seeking and using information about CVI that might influence their school SEND policies. We carried out a structured review of all each school’s publicly available SEND policy documentation pre-randomisation and at follow up. Documents assessed where available were: SEND Policy; Supporting children with medical needs policy; Accessibility plan; Behaviour policy; Anti-bullying policy and Inclusion policy. In some schools Inclusion and Anti-bullying policies were included within the school’s behaviour policy.

Within these documents we looked for evidence for: changes to classroom layout; classroom clutter reduction; how CVI might be identified within an individual child; change in referrals processes indicating a focus on sensory needs; changes in how sensory needs are identified and met; support services employed; changes to teaching methods or approaches to children; references in documents to vision/CVI or sensory support. In addition, we examined COVID related policy changes to see if it was possible to differentiate physical classroom changes that occurred due to the Intervention or due to COVID directives from the government.

In 3 (1 intervention and 2 control schools) of the 5 schools there was no difference in the publicly available documents reviewed from pre-randomisation to follow up. Changes were noted in 1 control school where the 2021-2022 SEND policy and report document contained much more detail than the 2019-2020 policy for example:

***“Paragraph 5.7 Adaptations to the curriculum and learning environment.*** *We make the following adaptations to ensure all pupils’ needs are met: Using recommended aids, such as laptops, coloured overlays, visual timetables, larger font, etc. Differentiating our teaching, for example, giving longer processing times, pre-teaching of key vocabulary, reading* *instructions aloud, etc.”* *SCHOOL 1*

In the intervention school, although no changes were recorded within SEND documents. It was noted that in revised 2020 documents the font was standardised to either Tahoma or Arial, compared to a mix of fonts including Times New Roman in 2019 documents. The change reflects the responses to interview questions about policy changes within the school, although as seen below it appears that the changes are due both to the CVI intervention and a recent school inspection.

*“…as a result of like I say the CVI and the research on working memory and everything else that’s going around in the school we are changing our expectations around the school in terms of displays and how worksheets are formatted, and also that expectation of don’t just gloss over things with a child, don’t just shrug and go oh well yes. So those are the three main things in policy change, it will be what classrooms should look like, that’s something that we’re very… I am very keen to keep, is that decluttered classroom.” SCHOOL 6*

*“I can’t remember, my head came down to one, and as a certain size, and I think as school we were guilty of [..] using fancy Disney fonts and things, things like that because they thought they were attractive to the children, and actually it just caused confusion around the classroom, and it was something that an inspector picked us up on, was the variety of fonts within the classroom. So it’s led to the whole school having a policy on the type on font, the size of font…” SCHOOL 6 Teacher*

Qualitative interviews enabled us to discover if staff themselves thought there were any policy changes and to discover any changes not publicly available or that were in the process of being introduced. Changes reported included format and layout of displays, presentations, and homework:

*“.our maths policy was changed due to the way that our PowerPoints within our lessons. Our homework policy has also changed…the homework policy is the fonts that are used on the sheets of paper, the way that the calculations are shown on the document, we’re only allowed to have a set amount of questions on the page. It’s English the sentences have bigger line spaces between them, just to make it clearer and just to give a bit more clarity for the children, particularly in key stage one.”* *SCHOOL 3*

*“Yeah, in terms of our presentation display documents that’s now a whole school policy, so not just within classrooms but across the school the way that displays should look with things not going above… outside the border of our display, the type of text you use, the size of fonts you use. So yeah that has a whole school policy change.*” *SCHOOL 6*

## Photographic ratings of classroom clutter

schools were asked to take photographs of each wall in each study classroom so that an assessment of changes to ‘clutter’ could be made using a clutter rating scale. We asked for the photographs at 3 timepoints: prior to randomisation (years 3, 4 and 5) when the schools opened fully in September 2020 (years 4, 5 and 6) and at follow up in March 2021 (years 4, 5 and 6). Staff were sent detailed instructions with a photographic illustration of what was required. As part of the process evaluation questions about acceptability of trial processes, we asked staff for their thoughts about this procedure.

Most schools found the process straightforward to complete, and while some schools understood the purpose of taking the photographs:

*“Absolutely, you can see certainly when you’re looking at decluttering the work areas and things like that, you’re looking for before and after aren’t you?” SCHOOL 6*

Others would have like to know more about why we asked for them

*“I am quite intrigued as to the results of this study because I have been taking photographs of classrooms for example, so I am still not aware of why I have had to do that, and what the processes are behind that. Because being in the control group we haven’t had to change anything, whereas presumably if we were in a different… the other group we might have been asked to make certain changes, and then it would have been clearer maybe what the photographs were used for.” SCHOOL 2*

For some staff, the process led them to reflect on the appearance of their classroom. The quotes below are from 2 of the control schools.

*“And a few of [teachers] said, ‘Well I was just about to put the display in,’ and I [headteacher] said, ‘This is not what this is about, this is the moment, I don’t want you doing anything for me, I want it to be actually this is part of a study this is real life. If everyone jazzes up what they have done because I am taking a photograph it’s not real.’ It was unfortunate that people were doing displays the week after, but that’s life as far as I’m concerned actually. I wanted it to be a this is our classrooms, not a here we have jollied one up for you.” SCHOOL 5*

*“I would say just from the fact that a teacher was coming around to take photos of our classroom displays etc, it made us all really look and focus more on our displays and what actually they look like, taking a picture.” SCHOOL 1*

*“But interesting that when we took the pictures people were like oh God is that really what my classroom looks like from where they sit? But we don’t know if that’s a good or a bad thing still. But they are quite busy’” SCHOOL 1*

***Conclusions and recommendations***

Overall, schools that continued participation in the feasibility study were happy with the intervention and trial processes. In terms of the intervention, they found the pack of resources useful, and liked being able to choose what to engage with. Decluttering classrooms was most popular, followed by changes in works sheets and PowerPoint presentations along with the animal stories for use during lessons.

schools with an enthusiastic leader of the project found it easier and staff appreciated being given dedicated time to engage with the resources and make changes. Delivering the pack during the summer was suggested as a way to improve acceptability of decluttering as this was by far the most controversial part of the intervention.

school key contacts and staff alike all indicated that more in person visits from the study team to explain the intervention and study measures would improve motivation for both.

All those interviewed (parents and school staff) indicated that onsite vision assessments would be preferable and making the referral process as simple as possible would help children access the first steps in an assessment for CVI. Parents felt positive about the vouchers for filling out questionnaires and taking part in interviews and suggested that this was a way to increase participation.

One head teacher suggested including a timetable for study participation to be included early on for managing expectations of the processes.

**Some very early recommendations for future trial designs:**

- Consider the timing of recruitment and delivery of the intervention to maximise the intervention period and teacher classroom set up.
- Consider study team visits to explain trial processes and maintain child completed questionnaire confidentiality. Initiation visits and perhaps follow up contacts (phone, video or in person visits) during course of study.
- Review pack contents with additional information stories and perhaps include non-fiction examples too.
- Onsite vision assessments are preferred by head teachers, school staff and parents.
- Maintain reimbursement for teacher time and vouchers for parent participation and any interviews
- Online data collection preferred and liked by parents.
- Benefits of ‘interim communication’ from study team to parents.

**References**

1. Moore G AS, Barker M, Bond L, Bonell C, Hardeman W, Moore L, O’Cathain A, Tinati T, Wight D, Baird J. Process evaluation of complex interventions: Medical Research Council guidance. MRC Population Health Science Research Network, London, 2014 Process evaluation of complex interventions: Medical Research Council guidance. London: MRC Population Health Science Research Network; 2014.

2. Gale RC, Wu J, Erhardt T, et al. Comparison of rapid vs in-depth qualitative analytic methods from a process evaluation of academic detailing in the Veterans Health Administration. *Implementation Science*. 2019;14(1):11.

3. Moore GF, Audrey S, Barker M, et al. Process evaluation of complex interventions: Medical Research Council guidance. 2015;350.

4. Schwarzer R, Schmitz GS, Daytner GT. The teacher self-efficacy scale. 1999.

5. Pilon-Kamsteeg F, Dekker-Pap MJ, de Wit GC, et al. CVI Experience toolbox: Simulation of visual processing difficulties. *British Journal of Visual Impairment*. 2019;37(3):248-57.
