# Supplementary material for: A school-based intervention to improve mental health outcomes for children with cerebral visual impairment (CVI): feasibility cluster randomised trial
Source: Pilot Feasibility Stud. 2025 Mar 3;11:24. doi: 10.1186/s40814-025-01603-x (PMC11874832; doi:10.1186/s40814-025-01603-x)
Supplement: Supplementary file 2 — Supplementary Material 2. Table 2.a: Baseline assessment data from Children, Teachers and Parents, for all and by Special Education Needs (SEN) status. Supplementary table 2b: Mean (95% CI) change in questionnaire score (follow up - baseline) for participants with scores at both timepoints, by study arm and by SEN status [file 40814_2025_1603_MOESM2_ESM.docx]

**CVI Feasibility crct Supplementary file Table 2.a:
Baseline assessment data from Children, Teachers and Parents, for all and by Special Education Needs (SEN) status**

|  | **Total**  **(N = 979)** | **Percent** | **Mean, (SD)** | **Median [IQR]** | **Range Min-Max** |  |
| --- | --- | --- | --- | --- | --- | --- |
|  |  |  |  |  |  |  |
| **Baseline Child PEDSQL physical function score (All Children)** | 898 | 91.7% | 76.6 ( 17.7) | 81.3 [65.6, 90.6] | 0.0 - 100.0 |  |
| *Missing* | 81 | 8.3% |  |  |  |  |
|  |  |  |  |  |  |  |
| Baseline Child PEDSQL physical function score (Has SEN) | 133 | 84.2% | 66.5 ( 22.0) | 71.4 [50.0, 84.4] | 0.0 - 100.0 |  |
| *Missing* | 25 | 15.8% |  |  |  |  |
|  |  |  |  |  |  |  |
| Baseline Child PEDSQL physical function score (No SEN) | 765 | 93.2% | 78.4 ( 16.2) | 81.3 [68.8, 90.6] | 15.6 - 100.0 |  |
| *Missing* | 56 | 6.8% |  |  |  |  |
|  |  |  |  |  |  |  |
| **Baseline Child PEDSQL Social function score (All Children)** | 896 | 91.5% | 74.2 ( 22.2) | 80.0 [61.3, 90.0] | 0.0 - 100.0 |  |
| *Missing* | 83 | 8.5% |  |  |  |  |
|  |  |  |  |  |  |  |
| Baseline Child PEDSQL Social function score (Has SEN) | 132 | 83.5% | 64.0 ( 27.2) | 70.0 [45.0, 85.0] | 0.0 - 100.0 |  |
| *Missing* | 26 | 16.5% |  |  |  |  |
|  |  |  |  |  |  |  |
| Baseline Child PEDSQL Social function score (No SEN) | 764 | 93.1% | 75.9 ( 20.7) | 80.0 [65.0, 92.5] | 0.0 - 100.0 |  |
| *Missing* | 57 | 6.9% |  |  |  |  |
|  |  |  |  |  |  |  |
| **Baseline Child PEDSQL Emotional function score (All Children)** | 897 | 91.6% | 63.3 ( 23.3) | 65.0 [45.0, 80.0] | 0.0 - 100.0 |  |
| *Missing* | 82 | 8.4% |  |  |  |  |
|  |  |  |  |  |  |  |
| Baseline Child PEDSQL Emotional function score (Has SEN) | 133 | 84.2% | 56.9 ( 24.6) | 55.0 [40.0, 75.0] | 0.0 - 100.0 |  |
| *Missing* | 25 | 15.8% |  |  |  |  |
|  |  |  |  |  |  |  |
| Baseline Child PEDSQL Emotional function score (No SEN) | 764 | 93.1% | 64.4 ( 22.9) | 65.0 [50.0, 83.1] | 0.0 - 100.0 |  |
| *Missing* | 57 | 6.9% |  |  |  |  |
|  |  |  |  |  |  |  |
| **Baseline Child PEDSQL School function score (All Children)** | 896 | 91.5% | 68.6 ( 22.0) | 75.0 [55.0, 85.0] | 0.0 - 100.0 |  |
| *Missing* | 83 | 8.5% |  |  |  |  |
|  |  |  |  |  |  |  |
| Baseline Child PEDSQL School function score (Has SEN) | 132 | 83.5% | 55.0 ( 24.6) | 55.0 [ 40.0, 75.0] | 0.0 - 100.0 |  |
| *Missing* | 26 | 16.5% |  |  |  |  |
|  |  |  |  |  |  |  |
| Baseline Child PEDSQL School function score (No SEN) | 764 | 93.1% | 71.0 ( 20.7) | 75.0 [ 57.5, 85.0] | 5.0 - 100.0 |  |
| *Missing* | 57 | 6.9% |  |  |  |  |
|  |  |  |  |  |  |  |
| **Baseline Child PEDSQL Psychosocial Health score (All Children)** | 898 | 91.7% | 68.7 ( 19.5) | 71.7 [56.7, 83.3] | 0.0 - 100.0 |  |
| *Missing* | 81 | 8.3% |  |  |  |  |
|  |  |  |  |  |  |  |
| Baseline Child PEDSQL Psychosocial Health score (Has SEN) | 133 | 84.2% | 58.5 ( 22.0) | 61.7 [46.7, 75.0] | 0.0 - 100.0 |  |
| *Missing* | 25 | 15.8% |  |  |  |  |
|  |  |  |  |  |  |  |
| Baseline Child PEDSQL Psychosocial Health score (No SEN) | 765 | 93.2% | 70.4 ( 18.5) | 73.3 [58.3, 85.0] | 6.7 - 100.0 |  |
| *Missing* | 56 | 6.8% |  |  |  |  |
|  |  |  |  |  |  |  |
| **Baseline Child PEDSQL generic core score (All Children)** | 893 | 91.2% | 72.6 ( 16.8) | 75.0 [62.4, 85.4] | 7.1 - 100.0 |  |
| *Missing* | 86 | 8.8% |  |  |  |  |
|  |  |  |  |  |  |  |
| Baseline Child PEDSQL generic core score (Has SEN) | 131 | 82.9% | 63.1 ( 18.9) | 66.2 [48.7, 76.0] | 7.1 - 99.0 |  |
| *Missing* | 27 | 17.1% |  |  |  |  |
|  |  |  |  |  |  |  |
| Baseline Child PEDSQL generic core score (No SEN) | 762 | 92.8% | 74.3 ( 15.9) | 77.0 [64.5, 86.9] | 15.5 - 100.0 |  |
| *Missing* | 59 | 7.2% |  |  |  |  |
|  |  |  |  |  |  |  |
| **Teacher SDQ Total Difficulties Score- BL (All Children)** | 920 | 94.0% | 6.7 ( 6.7) | 5.0 [ 1.0, 10.0] | 0.0 - 33.0 |  |
| *Missing* | 59 | 6.0% |  |  |  |  |
|  |  |  |  |  |  |  |
| Teacher SDQ Total Difficulties Score- BL (Has SEN) | 150 | 94.9% | 14.9 ( 7.1) | 14.0 [10.0, 21.0] | 0.0 - 30.0 |  |
| *Missing* | 8 | 5.1% |  |  |  |  |
|  |  |  |  |  |  |  |
| Teacher SDQ Total Difficulties Score- BL (No SEN) | 770 | 93.8% | 5.1 ( 5.4) | 4.0 [ 1.0, 7.0] | 0.0 - 33.0 |  |
| *Missing* | 51 | 6.2% |  |  |  |  |
|  |  |  |  |  |  |  |
| **Teacher SDQ Internalising Problems - BL (All Children)** | 920 | 94.0% | 3.0 ( 3.5) | 2.0 [ 0.0, 5.0] | 0.0 - 19.0 |  |
| *Missing* | 59 | 6.0% |  |  |  |  |
|  |  |  |  |  |  |  |
| Teacher SDQ Internalising Problems - BL (Has SEN) | 150 | 94.9% | 6.1 ( 3.9) | 6.0 [ 3.0, 8.0] | 0.0 - 17.0 |  |
| *Missing* | 8 | 5.1% |  |  |  |  |
|  |  |  |  |  |  |  |
| Teacher SDQ Internalising Problems - BL (No SEN) | 770 | 93.8% | 2.4 ( 3.1) | 1.0 [ 0.0, 4.0] | 0.0 - 19.0 |  |
| *Missing* | 51 | 6.2% |  |  |  |  |
|  |  |  |  |  |  |  |
| **Teacher SDQ Externalising Problems - BL (All Children)** | 920 | 94.0% | 3.7 ( 4.3) | 2.0 [ 0.0, 6.0] | 0.0 - 20.0 |  |
| *Missing* | 59 | 6.0% |  |  |  |  |
|  |  |  |  |  |  |  |
| Teacher SDQ Externalising Problems - BL (Has SEN) | 150 | 94.9% | 8.8 ( 4.6) | 9.0 [ 5.0, 12.0] | 0.0 - 20.0 |  |
| *Missing* | 8 | 5.1% |  |  |  |  |
|  |  |  |  |  |  |  |
| Teacher SDQ Externalising Problems - BL (No SEN) | 770 | 93.8% | 2.7 ( 3.4) | 1.0 [ 0.0, 4.0] | 0.0 - 18.0 |  |
| *Missing* | 51 | 6.2% |  |  |  |  |
|  |  |  |  |  |  |  |
| **Teacher SDQ Impact Score- BL (All Children)** | 919 | 93.9% | 0.6 ( 1.3) | 0.0 [ 0.0, 0.0] | 0.0 - 6.0 |  |
| *Missing* | 60 | 6.1% |  |  |  |  |
|  |  |  |  |  |  |  |
| Teacher SDQ Impact Score- BL (Has SEN) | 150 | 94.9% | 2.1 ( 1.9) | 2.0 [ 0.0, 3.0] | 0.0 - 6.0 |  |
| *Missing* | 8 | 5.1% |  |  |  |  |
|  |  |  |  |  |  |  |
| Teacher SDQ Impact Score- BL (No SEN) | 769 | 93.7% | 0.3 ( 0.8) | 0.0 [ 0.0, 0.0] | 0.0 - 6.0 |  |
| *Missing* | 52 | 6.3% |  |  |  |  |
|  |  |  |  |  |  |  |
| **Teacher PedsQL™ Cognitive Functioning Scale - BL (All Children)** | 919 | 93.9% | 69.9 ( 27.4) | 75.0 [50.0,100.0] | 0.0 - 100.0 |  |
| *Missing* | 60 | 6.1% |  |  |  |  |
|  |  |  |  |  |  |  |
| Teacher PedsQL™ Cognitive Functioning Scale - BL (Has SEN) | 150 | 94.9% | 35.1 ( 22.9) | 31.3 [20.8, 50.0] | 0.0 - 100.0 |  |
| *Missing* | 8 | 5.1% |  |  |  |  |
|  |  |  |  |  |  |  |
| Teacher PedsQL™ Cognitive Functioning Scale - BL (No SEN) | 769 | 93.7% | 76.7 ( 22.6) | 75.0 [62.5,100.0] | 4.2 - 100.0 |  |
| *Missing* | 52 | 6.3% |  |  |  |  |
|  |  |  |  |  |  |  |
| **Teacher 5 Question CVI Count Always Often Answers - BL (All Children)** | 914 | 93.4% | 0.2 ( 0.6) | 0.0 [ 0.0, 0.0] | 0.0 - 5.0 |  |
| *Missing* | 65 | 6.6% |  |  |  |  |
|  |  |  |  |  |  |  |
| Teacher 5 Question CVI Count Always Often Answers - BL (Has SEN) | 149 | 94.3% | 0.7 ( 1.1) | 0.0 [ 0.0, 1.0] | 0.0 - 5.0 |  |
| *Missing* | 9 | 5.7% |  |  |  |  |
|  |  |  |  |  |  |  |
| Teacher 5 Question CVI Count Always Often Answers - BL (No SEN) | 765 | 93.2% | 0.1 ( 0.4) | 0.0 [ 0.0, 0.0] | 0.0 - 5.0 |  |
| *Missing* | 56 | 6.8% |  |  |  |  |
|  |  |  |  |  |  |  |
| **Baseline Parent SDQ Difficulties Impact Score (All Children)** | 190 | 19.4% | 0.9 ( 1.9) | 0.0 [ 0.0, 0.0] | 0.0 - 10.0 |  |
| *Missing* | 789 | 80.6% |  |  |  |  |
|  |  |  |  |  |  |  |
| Baseline Parent SDQ Difficulties Impact Score (Has SEN) | 18 | 11.4% | 2.9 ( 2.6) | 2.5 [ 1.0, 5.0] | 0.0 - 10.0 |  |
| *Missing* | 140 | 88.6% |  |  |  |  |
|  |  |  |  |  |  |  |
| Baseline Parent SDQ Difficulties Impact Score (No SEN) | 172 | 21.0% | 0.6 ( 1.6) | 0.0 [ 0.0, 0.0] | 0.0 - 10.0 |  |
| *Missing* | 649 | 79.0% |  |  |  |  |
|  |  |  |  |  |  |  |
| **Parent 5 Question CVI Count Always Often Answers - BL (All Children)** | 189 | 19.3% | 0.2 ( 0.7) | 0.0 [ 0.0, 0.0] | 0.0 - 5.0 |  |
| *Missing* | 790 | 80.7% |  |  |  |  |
|  |  |  |  |  |  |  |
| Parent 5 Question CVI Count Always Often Answers - BL (Has SEN) | 18 | 11.4% | 1.2 ( 1.5) | 0.5 [ 0.0, 2.0] | 0.0 - 5.0 |  |
| *Missing* | 140 | 88.6% |  |  |  |  |
|  |  |  |  |  |  |  |
| Parent 5 Question CVI Count Always Often Answers - BL (No SEN) | 171 | 20.8% | 0.1 ( 0.5) | 0.0 [ 0.0, 0.0] | 0.0 - 4.0 |  |
| *Missing* | 650 | 79.2% |  |  |  |  |
|  |  |  |  |  |  |  |
| **Parent HRQL Summary BL Score (All Children)** | 187 | 19.1% | 86.1 ( 20.5) | 98.2 [76.8,100.0] | 7.1 - 100.0 |  |
| *Missing* | 792 | 80.9% |  |  |  |  |
|  |  |  |  |  |  |  |
| Parent FI Parent HRQL Summary BL Score (Has SEN) | 18 | 11.4% | 68.9 ( 24.6) | 68.8 [50.0, 92.9] | 17.3 - 100.0 |  |
| *Missing* | 140 | 88.6% |  |  |  |  |
|  |  |  |  |  |  |  |
| Parent FI Parent HRQL Summary BL Score (No SEN) | 169 | 20.6% | 87.9 ( 19.2) | 98.2 [80.4,100.0] | 7.1 - 100.0 |  |
| *Missing* | 652 | 79.4% |  |  |  |  |
|  |  |  |  |  |  |  |
| **Parent FI Parent Family Functioning Summary BL Score (All Children)** | 186 | 19.0% | 84.2 ( 22.9) | 100.0[71.9,100.0] | 0.0 - 100.0 |  |
| *Missing* | 793 | 81.0% |  |  |  |  |
|  |  |  |  |  |  |  |
| Parent FI Parent Family Functioning Summary BL Score (Has SEN) | 18 | 11.4% | 70.7 ( 21.6) | 71.9 [50.0, 93.8] | 37.5 - 100.0 |  |
| *Missing* | 140 | 88.6% |  |  |  |  |
|  |  |  |  |  |  |  |
| Parent FI Parent Family Functioning Summary BL Score (No SEN) | 168 | 20.5% | 85.6 ( 22.7) | 100.0[75.0,100.0] | 0.0 - 100.0 |  |
| *Missing* | 653 | 79.5% |  |  |  |  |
|  | | | | | | |

**CVI Feasibility crct Supplementary table 2b:
 Mean (95% CI) change in questionnaire score (follow up - baseline) for participants with scores at both timepoints, by study arm and by SEN status**

| **Variable** | **Total**  N | **Overall**  **Mean, (CI)** | **Arm 1 N** | **Arm 1**  **Mean, (CI)** | **Arm 1 SEN N** | **Arm 1 SEN**  **Mean, (CI)** | **Arm 2 N** | **Arm 2**  **Mean , (CI)** | **Arm 2 SEN N** | **Arm 2 SEN Mean, (CI)** |
| --- | --- | --- | --- | --- | --- | --- | --- | --- | --- | --- |
|  |  |  |  |  |  |  |  |  |  |  |
| **Child PEDSQL physical function** | **341** | **-0.5**  **( -2.3, 1.3)** | **167** | **2.6**  **( -0.1, 5.2)** | **32** | **4.1**  **( -2.8, 10.9)** | **174** | **-3.5**  **( -6.0, -1.0)** | **33** | **-1.3**  **( -9.9, 7.2)** |
|  |  |  |  |  |  |  |  |  |  |  |
| **Child PEDSQL Social function** | **339** | **1.9**  **( -0.5, 4.3)** | **167** | **4.7**  **( 1.1, 8.2)** | **32** | **8.9 ( -3.0, 20.8)** | **172** | **-0.8**  **( -4.1, 2.5)** | **32** | **-1.0**  **(-11.6, 9.6)** |
|  |  |  |  |  |  |  |  |  |  |  |
| **Child PEDSQL Emotional function** | **338** | **0.4**  **( -2.2, 3.0)** | **166** | **5.8**  **( 2.0, 9.5)** | **31** | **11.9 ( 1.7, 22.1)** | **172** | **-4.7**  **( -8.1, -1.4)** | **32** | **-9.0**  **(-16.8, -1.2)** |
|  |  |  |  |  |  |  |  |  |  |  |
| **Child PEDSQL School function** | **338** | **-0.2**  **( -2.5, 2.1)** | **167** | **1.8**  **( -1.5, 5.1)** | **32** | **7.7 ( -2.0, 17.4)** | **171** | **-2.1**  **( -5.4, 1.1)** | **31** | **-1.1**  **( -9.8, 7.6)** |
|  |  |  |  |  |  |  |  |  |  |  |
| **Child PEDSQL Psychosocial Hemalth** | **339** | **0.7**  **( -1.3, 2.6)** | **167** | **3.9**  **( 1.1, 6.8)** | **32** | **9.0 ( -0.5, 18.4)** | **172** | **-2.5**  **( -5.2, 0.1)** | **32** | **-3.8**  **(-11.4, 3.8)** |
|  |  |  |  |  |  |  |  |  |  |  |
| **Child PEDSQL generic core score** | **337** | **-0.8**  **( -2.5, 0.9)** | **166** | **2.5**  **( 0.0, 5.0)** | **31** | **7.1**  **( -0.5, 14.8)** | **171** | **-3.9**  **( -6.1, -1.7)** | **31** | **-3.8**  **(-11.0, 3.3)** |
|  |  |  |  |  |  |  |  |  |  |  |
| **Teacher report Cognitive Functioning scores** | **151** | **0.8**  **(-3.0, 4.7)** | **60** | **2.8**  **(-3.1, 8.8)** | **12** | **-1.0**  **(-16.7, 14.6)** | **91** | **-0.5**  **(-5.7, 4.7)** | **15** | **-3.0**  **(-18.4, 12.3)** |
|  |  |  |  |  |  |  |  |  |  |  |
| **Teacher report SDQ total difficulties score** | **151** | **-0.4**  **(-1.3 , 0.5)** | **60** | **--1.0**  **(-2.3, 0.3)** | **12** | **-1.0**  **(-5.0, 2.8)** | **91** | **0.0**  **(-1.3, 1.2)** | **15** | **-1.7**  **(-4.9, 1.5)** |
|  |  |  |  |  |  |  |  |  |  |  |
| **Teacher report SDQ internalizing score** | **151** | **0.0**  **(-0.6, 0.6)** | **60** | **-0.3**  **(-1.1, 0.5)** | **12** | **-0.4**  **(-2.6, 1.8)** | **91** | **0.1**  **(-0.6, 0.9)** | **15** | **-0.9**  **(-2.7, 0.8)** |
|  |  |  |  |  |  |  |  |  |  |  |
| **Teacher report SDQ externalizing score** | **151** | **-0.4**  **(-0.9, 0.1)** | **60** | **-0.7**  **(-1.5, 0.1)** | **12** | **-0.7**  **(-3.7, 2.3)** | **91** | **-0.2**  **(-0.9, 0.5)** | **15** | **-0.7**  **(-2.7, 1.2)** |
|  |  |  |  |  |  |  |  |  |  |  |
| **Teacher report SDQ**  **impact score** | **151** | **0.0**  **(-0.2, 0.2)** | **60** | **-0.1**  **(-0.4, 0.1)** | **12** | **-0.7**  **(-1.7, 0.4)** | **91** | **0.0**  **(-0.2, 0.3)** | **11** | **0.0**  **(-0.9, 0.9)** |
|  |  |  |  |  |  |  |  |  |  |  |
| **Parent report FIM HRQoL summary score** | **53** | **-2.8**  **(-7.3, 1.7)** | **12** | **-5.4**  **(-17.3, 6.5)** | **1** | **-14.3** | **41** | **-2.0**  **(-7.0, 2.9)** | **2** | **-5.4**  **(-16.2, 5.5)** |
|  |  |  |  |  |  |  |  |  |  |  |
| **Parent report FIM family functioning score** | **53** | **-2.8**  **(-7.3, 1.7)** | **12** | **1.0**  **(-11.5, 13.6)** | **1** | **6.3** | **41** | **-1.8**  **(-7.2, 3.6)** |  | **-6.3**  **(-18.9, 6.4)** |
|  | | | | | | | | | | |

**Footnote: for Child-report PedsQL scales and Teacher-report Cognitive scales, higher score is better QoL or Cognitive ability, range for each is 0-100. For the SDQ Total Difficulties (range 0-40) and Impact scores (range 0-9 for teacher report, 0-15 for parent report), a higher score denotes more difficulties or examples of problematic behaviour.**
