# Supplementary material for: A school-based intervention to improve mental health outcomes for children with cerebral visual impairment (CVI): feasibility cluster randomised trial
Source: Pilot Feasibility Stud. 2025 Mar 3;11:24. doi: 10.1186/s40814-025-01603-x (PMC11874832; doi:10.1186/s40814-025-01603-x)
Supplement: Supplementary file 3 — Supplementary Material 3. Table S3a. Frequency of parent-reported healthcare resource use at baseline and follow-up. Table S3b. Descriptive statistics of healthcare and out-of-pocket costs reported by parent/carers at baseline and follow-up. Table S3c. Descriptive statistics of parent/carer time spent supporting children in their education or health. Table S3d. Descriptive statistics of calculated child utility scores, mapped from the PedsQL to the CHU9D. [file 40814_2025_1603_MOESM3_ESM.docx]

**Table S3a. Frequency of parent-reported healthcare resource use at baseline and follow-up**

|  |  | Baseline (N =189) | | Follow-up (N = 146) | |
| --- | --- | --- | --- | --- | --- |
|  | **Healthcare resource** | **Does not have**  **SEN support**  **N=171** | **Does have SEN support**  **N=18** | **Does not have SEN support**  **N=135** | **Does have SEN support**  **N=11** |
| Vision related | Hospital appointment for vision related issue | 9 | 12 | 29 | 1 |
|  | Optician | 117 | 14 | 71 | 6 |
|  | GP for vision related issue | 5 | 2 | 10 | 0 |
|  | Qualified Teacher of Children and Young People with Vision Impairment (QTVI) | 0 | 3 | 0 | 0 |
| Non-vision related | Hospital appointment (not vision related) | 45 | 18 | 33 | 2 |
|  | GP appointment (not vision related) | 164 | 20 | 90 | 3 |
|  | Paediatrician | 6 | 5 | 6 | 3 |
|  | Health visitor | 1 | 0 | 3 | 1 |
|  | A&E visit | 37 | 7 | 19 | 0 |
|  | Social worker | 8 | 5 | 3 | 1 |
|  | Inpatient hospital stay | 4 | 2 | 2 | 0 |
|  | Speech and language therapy | 3 | 2 | 6 | 2 |
|  | Hearing | 12 | 0 | 2 | 0 |
|  | Occupational therapy | 2 | 6 | 3 | 1 |
|  | Physiotherapy | 2 | 1 | 31 | 0 |
|  | CAMHS | 8 | 6 | 6 | 3 |
|  | Educational psychologist | 15 | 3 | 2 | 3 |
|  | Dentist | 229 | 34 | 155 | 8 |
|  | Police | 2 | 0 | 0 | 1 |

**Table S3b. Descriptive statistics of healthcare and out-of-pocket costs reported by parent/carers at baseline and follow-up**

|  |  | Baseline | | | Follow-up | | |
| --- | --- | --- | --- | --- | --- | --- | --- |
|  |  | **Does not have**  **SEN support**  N=171 | **Does have**  **SEN support**  N=18 | **All**  N=189 | **Does not have**  **SEN support**  N=135 | **Does have**  **SEN support**  N=11 | **All**  N=146 |
| Health and social care (£) | Mean | 345.74 | 1851.64 | 489.16 | 306.51 | 390.18 | 312.81 |
|  | Standard deviation | 637.22 | 2137.25 | 988.26 | 751.67 | 515.41 | 735.50 |
|  | Median | 154.00 | 1178.00 | 170.50 | 88.25 | 117.75 | 98.50 |
| Vision related only (£) | Mean | 28.67 | 383.86 | 95.86 | 54.55 | 25.00 | 50.00 |
|  | Standard deviation | 43.29 | 521.69 | 258.38 | 33.57 | 7.07 | 32.66 |
|  | Median | 12.50 | 120.00 | 16.00 | 55.00 | 25.00 | 35.00 |
| Out of pocket costs (£) | Standard deviation | 6.58 | 95.86 | 29.44 | 22.15 | 0.00 | 20.48 |
|  | SD | 36.14 | 258.38 | 254.24 | 142.45 | 0.00 | 137.07 |
|  | Median | 0.00 | 16.00 | 0.00 | 0.00 | 0.00 | 0.00 |

**Table S3c. Descriptive statistics of parent/carer time spent supporting children in their education or health**

|  | | Baseline | | | Follow-up | | |
| --- | --- | --- | --- | --- | --- | --- | --- |
|  |  | **Does not have**  **SEN support**  N=171 | **Does have**  **SEN support**  N=18 | **All**  N=189 | **Does not have**  **SEN support**  N=135 | **Does have**  **SEN support**  N=11 | **All**  N=146 |
| Days off work to care for child (any carer) | N (%) reporting taking time off | 99 (57.9) | 12 (66.7) | 111 (58.7) | 31 (23.0) | 2 (18.2) | 33 (22.6) |
|  | Mean | 1.58 | 5.50 | 1.95 | 0.73 | 1.27 | 0.77 |
|  | Standard deviation | 2.03 | 9.49 | 3.63 | 1.82 | 3.13 | 1.94 |
|  | Median | 1.00 | 2.00 | 1.00 | 0.00 | 0.00 | 0.00 |
| Hours supporting child’s learning (any carer) | N (%) reporting taking time off | 65 (38.01) | 9 (50.0) | 74 (39.2) | 68 (50.4) | 7 (63.6) | 75 (51.4) |
|  | Mean | 3.55 | 4.39 | 3.63 | 41.74 | 14.30 | 39.67 |
|  | Standard deviation | 16.91 | 12.50 | 16.51 | 134.14 | 26.16 | 129.33 |
|  | Median | 0.00 | 1.00 | 0.00 | 0.25 | 1.00 | 0.40 |

**Table S3d. Descriptive statistics of calculated child utility scores, mapped from the PedsQL to the CHU9D**

|  | Baseline | | | Follow-up | | |
| --- | --- | --- | --- | --- | --- | --- |
|  | **Does not have**  **SEN support**  N=798 | **Does have**  **SEN support**  N=125 | **All**  N=893 | **Does not have**  **SEN support**  N=261 | **Does have**  **SEN support**  N=60 | **All**  N=321 |
| Mean | 0.915 | 0.887 | 0.911 | 0.911 | 0.888 | 0.907 |
| Standard deviation | 0.058 | 0.069 | 0.060 | 0.053 | 0.066 | 0.056 |
| Median | 0.927 | 0.901 | 0.923 | 0.923 | 0.902 | 0.919 |
| Min | 0.682 | 0.706 | 0.682 | 0.734 | 0.728 | 0.728 |
| Max | 0.988 | 0.990 | 0.990 | 0.984 | 0.985 | 0.985 |
